# Occupational Allergic Sensitization Among Workers Processing King Crab (*Paralithodes camtschaticus*) and Edible Crab (*Cancer pagurus*) in Norway and Identification of Novel Putative Allergenic Proteins

**DOI:** 10.3389/falgy.2021.718824

**Published:** 2021-08-23

**Authors:** Marte R. Thomassen, Sandip D. Kamath, Berit E. Bang, Roni Nugraha, Shuai Nie, Nicholas A. Williamson, Andreas L. Lopata, Lisbeth Aasmoe

**Affiliations:** ^1^Department of Community Medicine, University of Tromsø The Arctic University of Norway, Tromsø, Norway; ^2^Department of Occupational and Environmental Medicine, University Hospital North Norway, Tromsø, Norway; ^3^Molecular Allergy Research Laboratory, College of Public Health, Medical and Veterinary Sciences, James Cook University, Douglas, QLD, Australia; ^4^Australian Institute of Tropical Health and Medicine, James Cook University, Townsville, QLD, Australia; ^5^Centre for Food and Allergy Research, Murdoch Childrens Research Institute, Melbourne, VIC, Australia; ^6^Department of Medical Biology, University of Tromsø The Arctic University of Norway, Tromsø, Norway; ^7^Department of Aquatic Product Technology, Faculty of Fisheries and Marine Science, Institut Pertanian Bogor University, Bogor, Indonesia; ^8^Melbourne Mass Spectrometry and Proteomics Facility, Bio21 Molecular Science & Biotechnology Institute, The University of Melbourne, Melbourne, VIC, Australia

**Keywords:** allergy, crab, ige antibody, hemocyanin, occupational asthma, proteomics, shellfish, tropomyosin

## Abstract

**Introduction:** Asthma and allergy occur frequently among seafood processing workers, with the highest prevalence seen in the crustacean processing industry. In this study we established for the first time the prevalence of allergic sensitization in the Norwegian king- and edible crab processing industry and characterized the IgE-reactive proteins.

**Materials and Methods:** Two populations of crab processing workers participated; 119 king crab and 65 edible crab workers. The investigation included information on work tasks and health through a detailed questionnaire. Allergic sensitization was investigated by crab-specific IgE quantification and skin prick tests (SPT) to four in-house prepared crab extracts; raw meat, cooked meat, raw intestines and raw shell. Allergen-specific IgE binding patterns were analyzed by IgE immunoblotting to the four allergen extracts using worker serum samples. Total proteins in crab SPT extracts and immunoblot-based IgE binding proteins were identified by mass spectrometric analysis.

**Results:** Positive SPTs were established in 17.5% of king- and 18.1% of edible crab workers, while elevated IgE to crab were demonstrated in 8.9% of king- and 12.2% of edible crab processing workers. There was no significant difference between the king and edible crab workers with respect to self-reported respiratory symptoms, elevated specific IgE to crab or SPT results. Individual workers exhibited differential IgE binding patterns to different crab extracts, with most frequent binding to tropomyosin and arginine kinase and two novel IgE binding proteins, hemocyanin and enolase, identified as king- and edible crab allergens.

**Conclusions:** Occupational exposure to king- and edible crabs may frequently cause IgE mediated allergic sensitization. Future investigations addressing the diagnostic value of crab allergens including tropomyosin and arginine kinase and the less well-known IgE-binding proteins hemocyanin and enolase in a component-resolved diagnostic approach to crab allergy should be encouraged.

## Introduction

According to the Food and Agriculture Organization about 59.51 million people were engaged in seafood and aquaculture production in 2018 (1). Several studies have shown that airway symptoms and asthma are prevalent among production workers in the seafood industry, with the highest prevalence found in crustacean processing (2–5). Symptoms may be caused by both irritative and immunological reactions to inhaled bioaerosols containing constituents of seafood (6–8). Snow crab has previously been implicated in cases of occupational asthma and allergy with reported prevalence of 15.8 and 14.9%, respectively, among production workers (9). Clinical symptoms analyzed in another study determined asthma-like symptoms in up to 50% of the workers in different plants, as well as other types of allergic reactions (rhinitis, conjunctivitis, skin reactions) in up to 42% of the workers (5). During processing of crustaceans, many different proteins are released into the air and may act as sensitizing agents (10). Some allergenic proteins are common denominators of the allergic reaction in the majority of workers that show allergy to crabs (11). If more than 50% of the allergic subjects react to the allergen, it is termed a major allergen (12). A major allergenic protein in crustaceans is a heat stable muscle protein tropomyosin (13–15). Tropomyosin is a highly cross-reactive pan-allergen among crustaceans, molluscs, insects and dust-mites (3, 16–18). More workers seem to be sensitized to tropomyosin from boiled crustacean extract as compared to raw crustaceans (19–21). Specific IgE to arginine kinase has, in combination with tropomyosin, has been suggested as central molecular markers for crustacean allergy and has been identified in inhalational exposure and sensitization among snow crab processing workers (22, 23). However, several other shellfish allergens have been identified in ingestion related allergies (24). To achieve a more specific diagnosis and better management of workers experiencing occupational health problems when processing crab, the characterization of the allergens that may cause allergic sensitization is crucial. The objective of this work was to determine the prevalence of elevated specific IgE to crab proteins as well as the prevalence of skin prick test (SPT) reactivity among crab processing workers to edible and king crab. Furthermore, we attempted to correlate these results to self-reported respiratory symptoms, asthma and allergy among sensitized crab processing workers. In addition, novel as well as known crab allergens were identified using molecular and proteomic approaches, associated with atopy among the crab processing workers, and may assist in developing improved diagnostics for occupational allergy to crabs.

## Materials and Methods

### Study Population

The population in this study has previously been investigated in a study of lung function and prevalence of respiratory symptoms (25). A total of 119 king crab (*Paralithodes camtschaticus*) workers from four plants, and 83 edible crab (*Cancer pagurus*) workers from one plant participated in one or more parts of this study. The inclusion criterion was their work in the processing plants at the time of the study. The study was conducted in two production seasons; from September 2009 to January 2010, and in September and October 2011. Written informed consent was obtained from all participants of this study. The study was conducted with the approval of the Regional Committee for Medical Research Ethics in Northern Norway (2009/1037/REK nord).

### Questionnaire

A questionnaire was distributed and completed by 119 king crab workers and 65 edible crab workers containing questions on personal background, health and occupational characteristics. This questionnaire was previously developed and published (25) as well as used in previous studies in the seafood industry, with minor modifications (26, 27). Self-reported doctor-diagnosed asthma and/or allergy were defined by positive answer to the questions: “*Do you have asthma/allergy?”* and “*was your asthma/allergy diagnosed by a doctor?”* Smoking habits were categorized as never smokers (persons who had never smoked regularly) or ever smokers (persons who were current or former smokers). Self-reported respiratory symptoms were defined as positive if the workers answered “yes” to one or more of the following questions; “*Have you experienced wheezing in the last 12 months?*” and “*Do you usually cough or hem in the morning?*” and “*Do you cough daily/almost daily for, on average, 3 months or more throughout the year?*”

### Serological Tests

Blood samples of 113 king crab workers and 78 edible crab workers were collected in Vacutainer separation tubes. The samples were centrifuged and stored refrigerated until arrival at the laboratory after 2–4 days, and stored at −70°C until further analyses in the department of Laboratory Medicine at the University Hospital of North Norway. The atopy status of the workers was established by quantifying specific IgE antibody to common inhalant allergens on inhalation panels (IP6 and IP7 containing birch, timothy, alternaria, cladosporium, cat, horse, dog, house dust mite, and rabbit) in addition to sensitization to crab (boiled crabmeat from *Cancer pagurus* code f23) and shrimp (f24) using the ImmunoCAP system (Thermo Scientific, Phadia AB, Uppsala, Sweden). The detection of specific IgE ≥ 0.35 kU/L on one or both of the inhalation panels was used as a positive result for atopy. Workers with specific IgE ≥ 0.35 kU/L to crab were defined as having elevated IgE to crab.

### Protein Extracts From Crabs Used for Allergen Analysis and Skin Prick Testing

Raw and cooked king crab and edible crab were purchased from crab processing plants. Four separate aqueous total protein extracts were generated for king crab and edible crab, respectively; raw meat, cooked meat, raw intestines, and proteins extracted from raw crab shells. Briefly, each component was blended with phosphate buffered saline (PBS), pH 7.4, 4°C, in a Waring blender for 90 s (1 min low and 30 s high intensity). The crab mass to buffer volume ratio was kept constant for each protein extract. The slurry was further stirred (Heidolph RZR 2100 Electronic) in a refrigerated room for 2 h, and then centrifuged at 10,000 g for 1 h to remove insoluble shell debris. The supernatant was centrifuged at 80,000 g for 1 h, and the protein content in this supernatant assayed using the Bradford method (28). These solutions were the final crab extracts. The protein concentrations in the final king crab extracts were 2.7, 0.5, 5.9, and 4.21 mg/ml in raw meat, cooked meat, intestine and shell extracts, respectively. The protein concentrations in the final edible crab extracts were 1.8, 2.5, 2.4, and 1.9 mg/ml in raw meat, cooked meat, intestine, and shell extracts, respectively. The extracts were stored at −70°C until further use.

### Skin Prick Test

Skin prick tests (SPT) were performed on 40 king crab workers and 83 edible crab workers. In-house generated crab extracts were used, king crab extracts on king crab production workers and edible crab extracts on edible crab production workers. Standard commercial SPT solutions for positive control (1% histamine) and negative control (0.9% saline) were used (Soluprick, ALK-Abellö AS, Denmark). The SPT was performed on the ventral forearm, and reactions were read after 15 min. Positive results were identified as a wheal of ≥3 mm in the presence of a positive control of ≥3 mm, and no response to the negative control.

### SDS-PAGE and Immunoblotting

SDS-PAGE and immunoblotting was performed as described previously (23, 29) to analyze the workers' serum IgE antibody binding patterns to proteins in the four different extracts for each crab species. Sera from the 10 king crab and 10 edible crab workers with the highest specific IgE to crab by ImmunoCAP (>0.35 kU/L) were selected for this analysis, corresponding to worker codes in **Tables 2, 3**. Briefly, 10 μg of the crab extracts were separated on a 12% SDS-PAGE gel and transferred to a polyvinylidene difluoride (PVDF) membrane using a semi-dry western blotting apparatus (BioRad, USA). After blocking the unbound regions of the membrane using 5% skimmed milk in phosphate buffered saline with 0.05% Tween-20 (PBS-T), the membrane was incubated with 1:10 diluted worker sera for 16 h at 4°C using a slot-blot apparatus (Idea scientific, UA). The blot was subsequently incubated with rabbit anti-human IgE antibody diluted 1:10,000 (Dako, USA) in 2% blocking buffer and goat anti-rabbit IgG-HRP (Promega, Australia). After washing the blots with PBS-T, the IgE binding was visualized based on enhanced chemiluminescence (ECL). The IgE binding was semi-quantified using densitometric analysis as described earlier (21). IgE binding intensity was categorized as low, medium or high binding by two individual measurements. The molecular weight and intensity of the IgE binding proteins were calculated using Quantity One software (BioRad, USA). Allergograms were generated as described earlier (21) to compare the IgE antibody binding patterns between the king crab and edible crab workers, and to various crab extracts (raw meat, cooked meat, shell, and intestines). To determine which proteins bind IgE from sensitized workers, the reactive bands were excised from the SDS-PAGE gels and analyzed using in-gel tryptic digestion and mass spectrometric analysis as described below (30, 31).

### Proteomic Analysis of Crab Extracts and IgE Binding Proteins Using LC-MS/MS Tandem Mass Spectrometry

The in-house prepared crab extracts as well as IgE binding protein isolated from SDS-PAGE gels were digested using trypsin (Sigma Aldrich, CA, USA) as described previously (32, 33). Tryptic-digested peptide samples were analyzed by liquid chromatography coupled to tandem mass spectrometry (LC-MS/MS). The LC system, Ultimate 3000 RSLC (Thermo Fisher Scientific, San Jose, CA) was equipped with an Acclaim Pepmap trap column (C18, 100 Å, 75 μm × 2 cm, Thermo Fisher Scientific, San Jose, CA) and an Acclaim Pepmap RSLC analytical column (C18, 100 Å, 75 μm × 50 cm, Thermo Fisher Scientific, San Jose, CA) at 50°C. The peptide mixture was loaded at an isocratic flow of 5 μL/min of 3% acetonitrile containing 0.05% trifluoroacetic acid for 5 min. The eluents used for the LC were water with 0.1% v/v formic acid and 5% v/v dimethyl sulfoxide (DMSO) for solvent A and acetonitrile with 0.1% v/v formic acid and 5% DMSO for solvent B. The gradient used (300 nL/min) was from 3% B to 22% B for 59 min, 22% B to 40% B in 10 min, 40% B to 80% B in 5 min, and maintained at 80% B for the final 5 min before equilibration for 9 min at 3% B prior to the next analysis.

The spray voltage, temperature of ion transfer tube and S-lens of the Q Exactive Plus Orbitrap mass spectrometer were set at 1.8 kV, 250°C and 50%, respectively. The full MS scans were acquired at m/z 375–1,400, a resolving power of 70,000, an AGC target value of 3.0 × 106 and a maximum injection time of 50 ms. The top 15 most abundant ions in the MS spectra was subjected to higher-energy collisional dissociation (HCD) at a resolving power of 17,500, AGC target value of 5 × 104, maximum injection time of 50 ms, isolation window of m/z 1.2 and normalized collision energy (NCE) of 30%. Dynamic exclusion of 30 s was enabled. Raw data was analyzed through Proteome Discoverer 2.3 (Thermo Fisher Scientific, San Jose, CA) using the Crustacean database (https://doi.org/10.25903/qdmh-4r06), Sequest search engine, trypsin as enzyme with maximum 2 missed cleavage, oxidation at methionine as variable modification, carbamidomethyl at cysteine as fixed modification, 10 ppm and 0.2 Da for MS and MS/MS mass tolerance. At least 2 peptides were required for each protein group.

### Statistics

Descriptive statistics are presented as mean or median with standard deviations for continuous variables, and frequencies with percentages for categorical variables. Single variable analyses were performed with Pearson Chi-squared/Fisher exact test and *t*-test. The statistical analyses were performed using IBM SPSS software package, version 24 where *p* < 0.05 were considered statistically significant.

## Results

### Questionnaire Outcomes

The demographics of the study group as well as work history and selected clinical data and smoking habits are shown in Table 1. The work force comprised of predominantly male workers and ever smokers (current or former smokers). The median length of employment was 1.6 years for king crab workers and 1.5 years for edible crab workers. Self-reported, doctor diagnosed asthma was reported in 5.0% of king crab workers and 3.1% of edible crab workers. The prevalence of self-reported, doctor diagnosed allergy was 19.3% among king crab workers and 10.8% among edible crab workers. Respiratory symptoms were reported in 36.6% of king crab workers and 29.2% of edible crab workers. There was a significantly increased percentage of ever smokers among king crab workers compared to edible crab workers. No other significant differences were found between the two groups.

**Table 1:** Demographics of the study population.

	**King crab workers (*n* = 119)**	**Edible crab workers (*n* = 83)**
*Participants (n)*		
Health examinations	113	83
Questionnaire	119	65
Age years, mean (SD)	38.9 (12.9)	35.6 (10.5)
Gender male (%)	66.7	68.1
Smoking ever^a^ (%)^*^	80.1	63.6
Employment, years (median)	1.6	1.5
Atopy^b^ (%)	29.2	30.8
Asthma^c^ (%)	5.0	3.1
Allergy^c^ (%)	19.3	10.8
Respiratory symptoms (%)	36.6	29.2
Crab IgE ≥ 0.35 kU/L (%)	8.9	12.2
Skin prick test positive^d^ (%)	17.5	18.1

* *Statistically significant difference between king crab and edible crab workers*.

a *Ever smokers-former and current smokers*.

b *Atopy: IgE ≥ 0.35 kU/L to at least one common inhalant allergen*.

c *Self reported doctor-diagnosed*.

d *Positive skin prick test to one or more of crab extracts- raw meat, cooked meat, shell, and intestines*.

### Atopy Status and Allergic Sensitization to Crabs

The prevalence of atopy was similar in both groups with 29.2% among king crab workers and 30.8% among edible crab workers (Table 1). Positive sensitization (workers with elevated IgE to crab and/or a positive SPT to crab) was established in 13 king crab workers and 17 edible crab workers. Table 2 lists the sensitized king crab workers and Table 3 the sensitized edible crab workers. Specific IgE to crab was established in 8.9% of king crab workers and 12.2% of edible crab workers. Positive SPT to one or several components of the crab was established in 17.5% of king crab workers and 18.1% of edible crab workers. Both SPT positive and positive specific IgE to crab was established in 12.5% of king crab workers and 9.6% of edible crab workers. Tables 2, 3 also demonstrates whether the sensitized workers reported respiratory symptoms, asthma or allergy, and if they were atopic (IgE ≥ 0.35 kU/L to at least one common inhalant allergen). Edible crab workers had a significantly higher prevalence of SPT positive reactions to shell and cooked crab compared to king crab workers. Most SPT-positive workers reacted to cooked crabmeat extracts, either alone or in combination with other extracts. The association between SPT, specific IgE to crab, self-reported respiratory symptoms, asthma, atopy, and allergy in king crab workers and edible crab workers is shown in Table 4. Atopy was significantly associated with positive SPT, specific IgE to crab, self-reported asthma and allergy. An association was also established between SPT and workers with elevated specific IgE to crab. Self-reported respiratory symptoms were significantly associated with self-reported allergy.

**Table 2:** Allergic symptoms and serum diagnostic of king crab processing workers.

**King crab worker no.^a^**	**Positive SPT to crab extracts^b^**	**Specific crab IgE (kU/L)^c^**	**Atopy^d^**	**General respiratory symptoms^e^**	**Self-reported asthma (A) or allergy (Al)^f^**
1	NT	0.55	–	N	–
2	–	3.31	+	MD	–
3	NT	0.59	+	Y	–
4	NT	2.62	+	Y	A/Al
5	C, I	2.39	+	N	–
6	R, C, I, S	1.76	+	Y	A/Al
7	R	0.89	+	MD	–
8	–	0.67	–	N	Al
9	C	6.61	+	Y	–
10	–	0.38	–	MD	–
11	I	0.18	+	Y	A/Al
12	R	0.09	–	N	–
13	R	0.08	–	Y	–

*SPT, Skin prick tests; specific IgE to crab, atopy, self-reported prevalence of respiratory symptoms, asthma, and allergy*.

a *Workers 1–10 in this table corresponds to worker 1–10 in the allergograms in **Figure 3***.

b *King crab extract; R, raw meat extract; C, cooked meat extract; I, intestinal extract; S, shell extract; –henegative to all extracts; NT, not tested*.

c *Commercial assay made from cooked crab meat using the ImmunoCAP system*.

d *Atopy status: Positive (+) or negative (–) IgE ≥ 0.35 kU/L to at least one common inhalant allergen*.

e *Y, yes; N, no; MD, missing questionnaire data*.

f *Answers from questionnaire: –, negative to both asthma and allergy; A, positive answer to asthma; Al, positive answer to allergy*.

**Table 3:** Allergic symptoms and serum diagnostic of edible crab processing workers.

**Edible crab worker no.^a^**	**Positive SPT to crab extracts^b^**	**Specific crab IgE (kU/L)^c^**	**Atopy^d^**	**General respiratory symptoms^e^**	**Self-reported asthma (A) or allergy (Al)^f^**
1	R, C, I, S	1.19	–	MD	MD
2	–	1.16	+	N	–
3	–	0.65	+	MD	MD
4	R, C, S	14.70	+	Y	–
5	R, C, S	5.41	+	Y	Al
6	C, S	0.63	–	N	–
7	R, C	4.32	+	N	–
8	R, C, I, S	3.84	+	MD	MD
9	R, C, I, S	11.70	+	Y	Al
10	C	105.00	+	Y	A
11	R, C, I	0.10	+	N	–
12	C	0.09	–	N	–
13	C, S	0.04	–	N	–
14	C	0.09	–	Y	–
15	S	0.07	+	N	–
16	C	0.18	–	N	–
17	C, I, S	NT	NT	MD	MD

*SPT, Skin prick tests; specific IgE to crab, atopy, self-reported prevalence of respiratory symptoms, asthma, and allergy*.

a *Workers 1–10 in this table corresponds to worker 1–10 in the allergograms in **Figure 4***.

b *Edible crab extract; R, raw meat extract; C, cooked meat extract; I, intestinal extract; S, shell extract; –henegative to all extracts; NT, not tested*.

c *Commercial assay made from cooked crab meat using the ImmunoCAP system*.

d *Atopy status: Positive (+) or negative (–) IgE ≥ 0.35 kU/L to at least one common inhalant allergen*.

e *Y, yes; N, no; MD, missing questionnaire data*.

f *Answers from questionnaire: –, negative to both asthma and allergy; A, positive answer to asthma; Al, positive answer to allergy*.

**Table 4:** Statistical associations (*p*-values) between skin prick test (SPT) reactivity, specific IgE by ImmunoCAP to crab and symptoms.

	**King crab**	**Edible crab**	**All workers**
**Associations with positive SPT**			
Asthma	0.085	0.317	0.090
Allergy	0.605	1.000	0.759
Atopy	0.002^†^	0.081^*^	0.001^*^^†^
Respiratory symptoms	0.424	0.756	0.469^*^
IgE to crab	0.001^†^	0.000^†^	0.000^*^^†^
**Associations with IgE to crab**			
Asthma	0.150	0.230	0.070
Allergy	0.369	0.635	0.308
Atopy	0.001^†^	0.001^†^	0.000^†^
Respiratory symptoms	1.000	0.484	0.662^*^
**Associations with respiratory symptoms**			
Asthma	0.031	0.083	0.004
Allergy	0.120^*^	0.000^†^	0.000^*^^†^
Atopy	0.072	0.603^*^	0.072^*^

*Analyses were performed on king crab workers and edible crab workers separately, and on all workers combined*.

*Associations were calculated using Fisher exact test, or χ^2^-test for n > 5*.

* *χ^2^-test was used*.

† *Statistically significant association was found (p < 0.05)*.

### Proteins and Putative Allergens in Crab SPT Extracts

**Figure 1:**
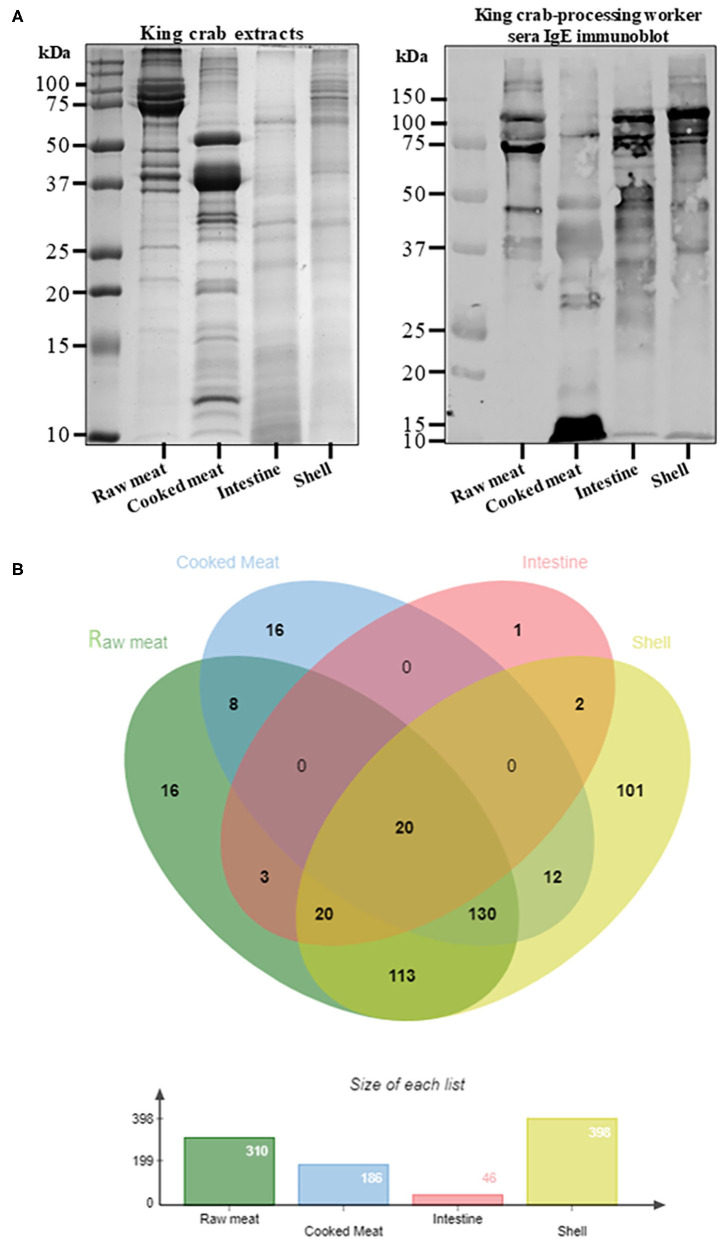
Protein profiling of king crab extracts. **(A)** SDS-PAGE separation of protein from raw meat, cooked meat, intestine and shell extracts, and immunoblotting using pooled serum from king crab processing worker to visualize IgE binding to different crab proteins. **(B)** Venn diagram showing total protein content using in-solution digest and subsequent mass spectrometric identification. The different overlapping regions indicate number of proteins that were commonly shared among two or more crab extracts. Detailed protein identification data is summarized in the online repository; Supplementary Table s10.

**Figure 2:**
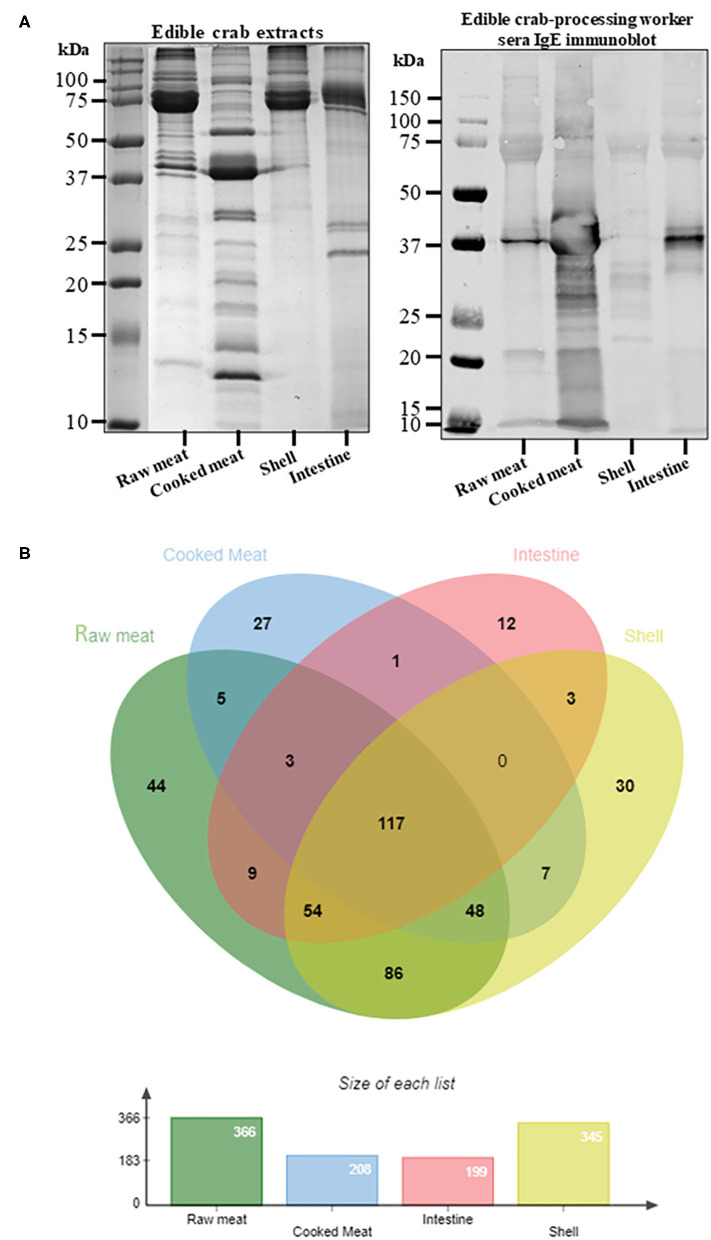
Protein profiling of edible crab extracts. **(A)** SDS-PAGE separation of protein from raw meat, cooked meat, intestine and shell extracts, and immunoblotting using pooled serum from edible crab processing worker to visualize IgE binding to different crab proteins. **(B)** Venn diagram showing total protein content using in-solution digest and subsequent mass spectrometric identification. The different overlapping regions indicate number of proteins that were commonly shared among two or more crab extracts. Detailed protein identification data is summarized in the online repository.

The protein content as well as the IgE binding patterns to the king and edible crab protein extracts were analyzed using SDS-PAGE and immunoblotting (Figures 1A, 2A). Abundant proteins visualized by intense bands were found in raw and cooked meat extracts as compared to shell and intestine extracts. IgE binding to crab proteins were mainly found to proteins higher than 25 kDa in size. A total of 442 proteins were identified in king crab extracts (raw meat-310, cooked meat-186, intestine-46, shell-398) and 446 proteins in edible crab extracts (raw meat-366, cooked meat-208, intestine-199, shell-345) (Figures 1B, 2B). In general, raw meat and shell extracts contained a higher number of proteins and the least number of proteins were identified in intestine extracts.

In king crab extracts, only 20 proteins were found common in all four extracts, including the allergen tropomyosin (Figure 1B, Supplementary Table 9). Interestingly, hemocyanin was predominantly found in the intestine extract, and shared with only raw meat and shell extracts. The cooked meat extract mainly contained proteins involved in muscle function or metabolism including known allergens such as tropomyosin, arginine kinase, myosin light chain, sarcoplasmic calcium binding protein, and triose phosphate isomerase. By contrast, intestine extract mainly contained enzymatic proteins such as trypsin, collagenolytic serine protease, and metalloendopeptidase, which were shared with the shell extract along with tropomyosin and hemocyanin, as shown by the data using mass spectrometric analysis. Interestingly, chitin deacetylase was found only in King crab shell extract.

In edible crab extracts, 117 proteins were commonly found in all four extracts, in contrast to 20 proteins in king crab. These included tropomyosin, arginine kinase, sarcoplasmic calcium binding protein, hemocyanin, and triose-phosphate isomerase (Figure 2B, Supplementary Table 9). Enolase and vitellogenin was found commonly in raw, intestine, and shell extracts and not in cooked meat extracts. Similar to king crab, edible crab intestine extract contained enzymatic proteins including cathepsins, catalase and chitinases. Interestingly, the cuticle protein family was identified in the shell and cooked meat extracts.

### IgE Antibody Binding Pattern to Crab Allergens

**Figure 3:**
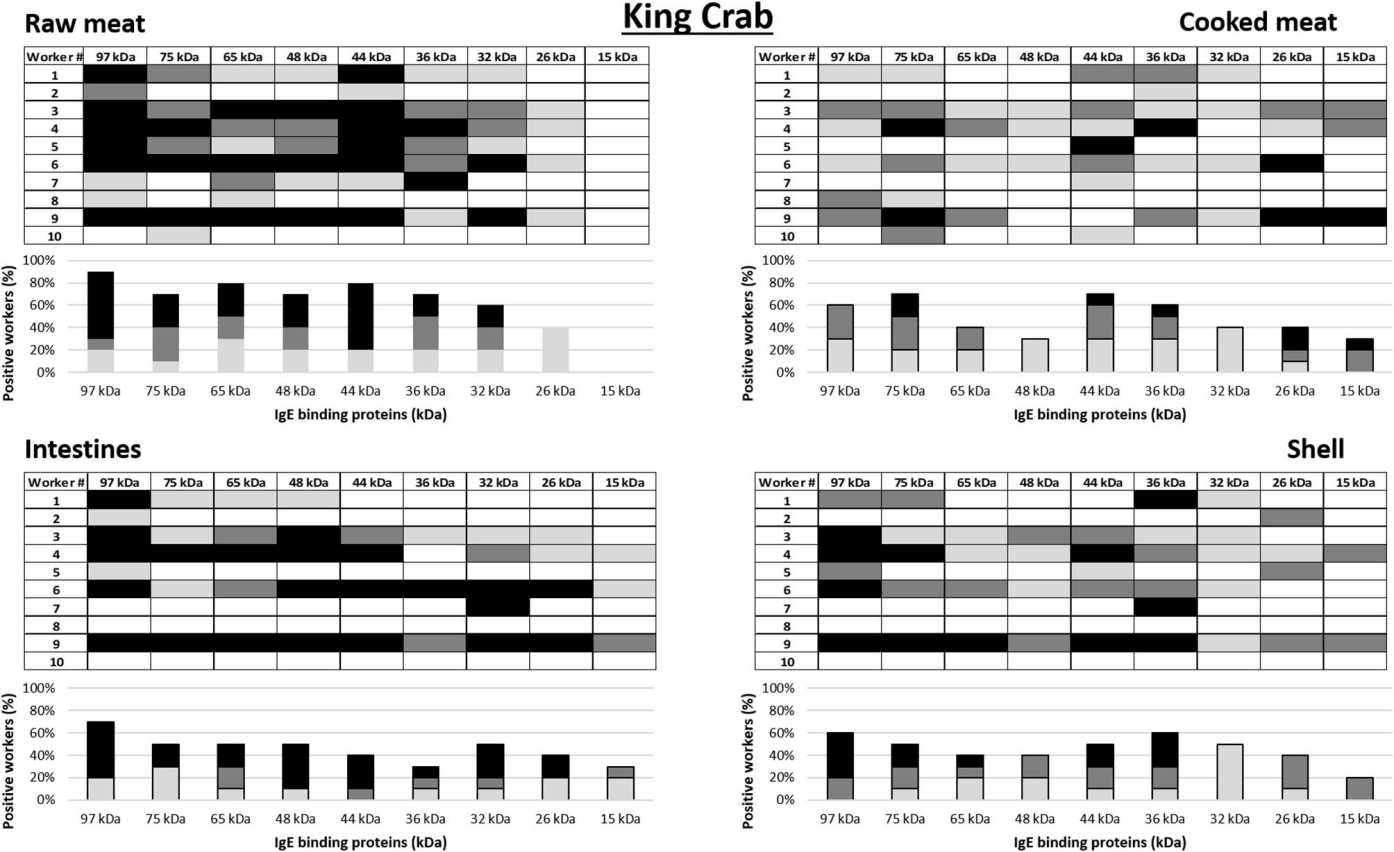
Allergograms of serum IgE binding to king crab proteins, including raw meat, cooked meat, raw intestines, and raw shell. Shadings correspond to high (black), medium (dark gray), and low (light gray) IgE binding to individual proteins shown in separate columns indicated by molecular weight (kDa).

**Figure 4:**
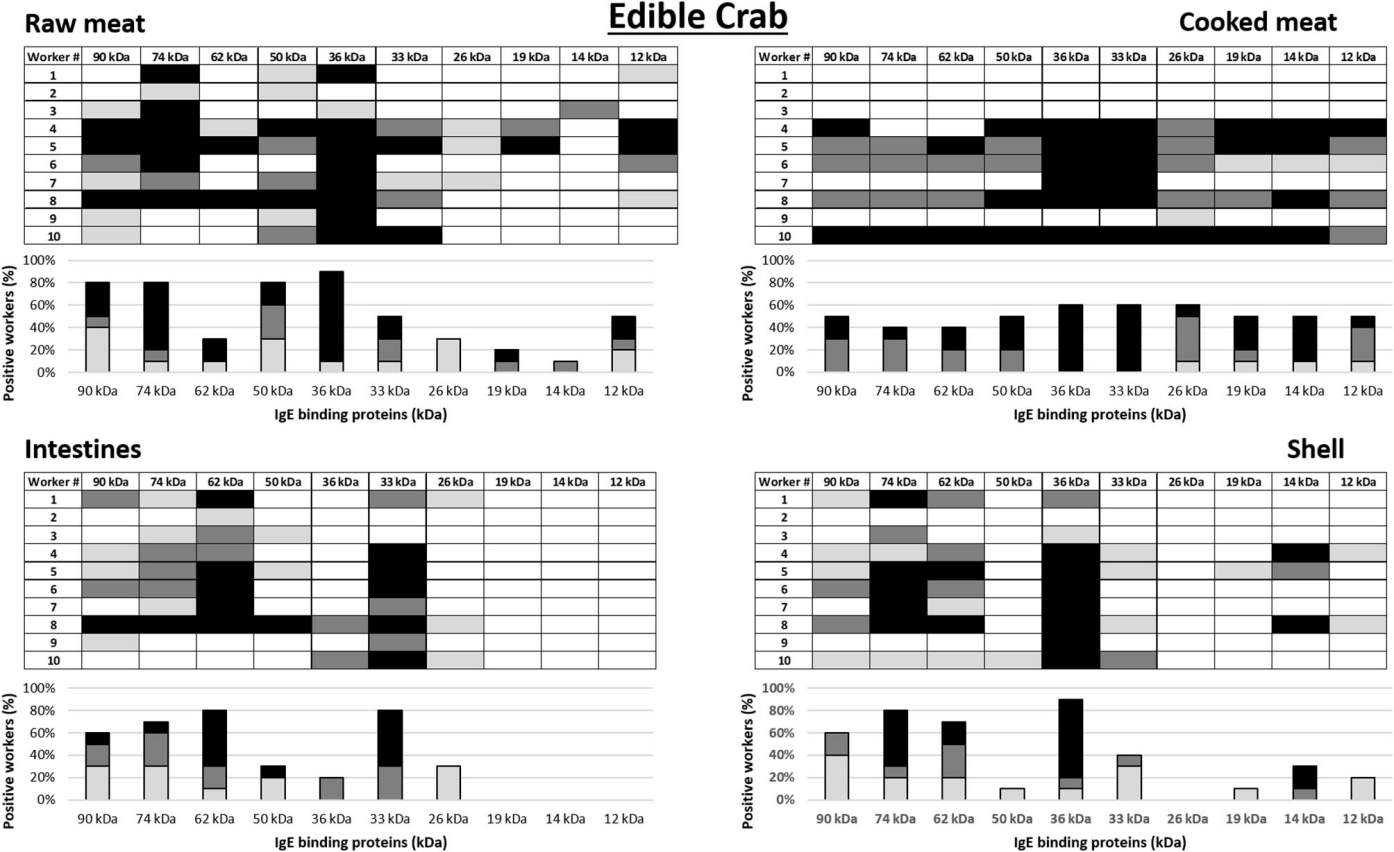
Allergograms of serum IgE binding to edible crab proteins, including raw meat, cooked meat, raw intestines, and raw shell. Shadings correspond to high (black), medium (dark gray), and low (light gray) IgE binding to individual proteins shown in separate columns indicated by molecular weight (kDa).

IgE antibody recognition of crab proteins from the raw and cooked meat, intestine and shell extracts were analyzed by immunoblotting using sera from the 10 king crab and 10 edible crab workers with the highest specific IgE to crab on immunoCAP (>0.35 kU/L) (worker 1–10 in Tables 2, 3 for king crab and edible crab workers, respectively). Allergograms were generated to compare the IgE recognition frequency and intensity to the most common IgE binding proteins in the immunoblots (Figures 3, 4 for king crab and edible crab workers, respectively). The IgE binding proteins contained in these SDS PAGE gel bands were identified using mass spectrometry (Supplementary Tables 1–8). Differential IgE binding to crab proteins was observed among the different crab extracts (Supplementary Figures 1A–H). Cooking of the crabmeat resulted in altered IgE binding patterns compared to raw meat extract. IgE binding was observed more frequently to higher molecular weight proteins in all extracts (larger than 26 kDa) compared to low molecular weight crab proteins. Individual workers showed different IgE binding to the various crab extracts, particularly to meat, intestine and shell extracts of both king and edible crabs. A high frequency of worker sera IgE binding was observed to crab proteins with an approximate molecular weight of 32–44 kDa and 65–75 kDa for both king and edible crabs. In king crab, strong IgE binding was observed to proteins in the raw meat and intestine extracts. In edible crab, IgE binding was observed frequently between 36 and 74 kDa, but no IgE binding was observed for proteins below 26 kDa in the intestine and shell extracts. King crab raw meat had demonstrated the highest frequency of IgE binding proteins as compared to edible crab raw meat.

Mass spectrometric analysis revealed that, IgE binding was observed to tropomyosin (~36 kDa) in all king and edible crab extracts near the 32–44 kDa region, as well as to the dimeric form at 75 kDa as shown previously (34, 35). IgE binding was also frequently observed to arginine kinase (~42 kDa) in all different crab extracts. Strong IgE binding was frequently observed to hemocyanin (~75 kDa) in the raw meat, intestine and shell extracts in both crab species. In addition, hemocyanin was identified in higher molecular weight bands suggesting IgE binding to high molecular weight oligomers. IgE binding was also observed to enolase (~47 kDa) in both groups of crab workers. It was found in king crab raw meat, intestine and shell extract, but only in edible crab raw meat extract. IgE binding was also found to known allergens such as sarcoplasmic calcium binding protein, vitellogenin, cuticle protein group and fructose bisphosphate aldolase, although less frequently.

### Allergic Sensitization and IgE Binding Patterns to Crab Proteins

When comparing the results of the SPT to the allergograms of allergen reactivity of the same worker, there are several similarities, but also some discrepancies. All workers with elevated specific IgE to crab extracts by ImmunoCap demonstrated also *in vitro* IgE binding to crab allergens using in-house immunoblots. Several workers who reacted to only some of the SPT extracts show IgE binding to all four extracts in the allergograms. King crab worker number 5 who did not report respiratory symptoms, asthma or allergy was SPT positive to cooked crabmeat and intestines and had IgE binding to several proteins in all four extracts. In contrast, in king crab worker number 2, an elevated IgE in the commercial test to crab was not reflected by respiratory symptoms, asthma, allergy or the SPT, and only a low binding intensity was identified to two proteins in raw meat and one in intestines in the in-house-immunoblot assay. Edible crab workers 2 and 3 had elevated specific IgE to crab by ImmunoCAP, but did not have positive SPT results, nor did they show any IgE binding to cooked crabmeat by immunoblotting. In contrast, edible crab worker number 10 had a high specific IgE to cooked edible crabmeat by ImmunoCAP, positive SPT to cooked meat extract, and strong binding to almost all proteins in the cooked meat extract by immunoblotting. This worker also showed strong IgE binding to proteins in the three other extracts, however SPT was negative to these extracts.

## Discussion

The aim of this study was to establish the prevalence of allergic sensitization among crab processing workers and identify the sensitizing allergens in these two different crab species. Furthermore, we explored the associations between positive IgE to crab proteins, positive SPT and reported respiratory symptoms.

Both king crab and edible crab workers were predominantly male with mean age of approximately 35 years, and with the mean time of employment being 1.5 years. Significantly more king crab workers were smokers compared to the edible crab workers. Atopic workers have been found to have an increased risk of developing IgE sensitization (36). In our study, 29.2% of king crab workers and 30.8% of edible crab workers were characterized as atopic. The workers reported a lower prevalence of asthma (5% in king crab and 3.1% in edible crab workers) compared to the general adult population in Norway where studies report 8–11.5% asthma prevalence (37, 38).

However, 36.6% of king crab workers and 29.3% of edible crab workers reported respiratory symptoms, which is higher than what has been found in previous studies including a population study in southern Norway (38) as well as studies in the snow crab industry (4, 39). Sensitization and upper respiratory symptoms have been found to precede the development of more serious health problems such as asthma and allergy (40, 41). The increased prevalence of respiratory symptoms among the crab processing workers may be precursors of more serious ill health caused by their work environment.

In the king crab plants participating in this study, most of the workers were processing raw crab. However, an open plant layout without shielding of the cooking process, resulted in exposure to bioaerosols from both raw and cooked production to all processing workers (42). In the edible crab industry however, most of the processing was performed on cooked crab, which was separated from the raw processing, limiting the number of exposed workers. Significantly higher levels of tropomyosin was found in edible crab processing compared to king crab processing, and the levels were highest during cooked edible crab processing (42). These differences in processing procedures may influence differences in sensitization to components of the bioaerosols. An exposure-response relationship between bioaerosol exposure and airway symptoms, occupational asthma and allergy has been described in various types of seafood industry (9, 43, 44). Inhalational exposure and sensitization to aerosolized food proteins is suggested to comprise a distinct form of food allergy proposed as “class 3 food allergy.” Sensitization through the inhalation route is rarely associated with symptoms after ingestion, although a few cases are described (45).

The prevalence of SPT positive workers were 17.5 and 18.1% in king crab and edible crab workers, respectively. This is lower than previous studies among snow crab processing workers (9, 46, 47) which may be due to differences in processing, exposure, use of personal protective equipment or the SPT extracts used. Due to lack of commercially available extracts for SPT, the in-house made extracts are used independently for each study, and any differences between the extracts would not be traceable. However, when the extracts are made from the allergen source that is most relevant to the exposure to workers, this will be optimal for determining if the crab is the causative agent for the observed sensitization. The high number of edible crab workers with positive SPT outcomes to cooked crab meat SPT extracts is supported by findings that heating shellfish increases the antibody affinity to tropomyosin (21, 48, 49) in addition to the fact that most edible crab processing workers handled cooked crab.

Our study highlighted the difference in protein and allergen composition in the different extracts as well as between two crab species. Using proteomic analysis, the presence of a unique set of crab proteins (including hemocyanin and enolase) was shown predominantly in the raw meat, intestine or shell components; all of which were not thermally processed, indicating the heat-labile nature of these proteins. Yet, immunoblot analysis with worker sera showed strong IgE binding to these proteins, and may play an important role in allergic sensitization among the crab workers. The abundance of heat stable allergen such as tropomyosin was found in all different protein extracts.

IgE binding analysis conducted in this study have shown that workers are sensitized to several and different proteins in the crabs. These reactivities do however not necessarily match with the results of the skin prick tests. A comparison of the allergograms of IgE reactivity between the same proteins in raw and cooked meat indicates a higher number of IgE binding proteins in the raw meat in both crab species. However, king crab processing workers demonstrated stronger IgE binding to the raw meat while edible crab processing workers had strong IgE binding to the cooked meat. The current study compared for the first time differential IgE binding analysis of two processed crab species as well as between raw meat, cooked meat and in particular intestine and crab shell extract that were used for the skin prick testing analysis. It was evident from the experimental data that the different processing methods as well as isolated crab sections resulted in altered IgE binding to the various allergens. As shown previously, tropomyosin and arginine kinase were identified in the raw and cooked meat, shell and intestines extracts. However, we have shown for the first time that hemocyanin is present in crab intestines and shell. It has been previously identified as an ingestion related allergen in crab roe (50) and shrimp as well as an inhalant cockroach allergen (51). Also, the known allergen enolase (52) previously identified in fish, was shown to be present in raw crabmeat and intestines. These putative crab allergens may have played a significant role in the inhalational sensitization of workers handling crab intestines on the processing lines. Enolase in fish was found to be a heat sensitive allergen (53, 54) and was found only in raw meat and raw intestines in our study. Hemocyanin has been found to be heat stable in shrimp (55–57), but was only identified in crab intestines and shell. These findings suggest that these allergens are more important as allergens in the work environment than in food consumption. As both king crab and edible crab's intestines are removed and crabs cooked during processing, these allergens are unlikely of major importance for ingestion induced seafood allergies. Chitin and its derivative chitosan, which is abundantly present in crab shells, has been indicated to play an important role in causing asthma and occupational allergy (58). Our study involved the use of aqueous buffer based crab extracts, which resulted in the exclusion of these potential components. Proteomic analysis of the extracts showed the presence of proteins such as chitinases and chitin deacetylases. Although IgE binding was not detected to these proteins in the crab extracts, these enzymes could potentially play a role in exacerbating allergic sensitization to crabs. Previous studies have indicated the presence of air-borne chitin particles in shellfish processing factories (42), and the effects of this exposure warrants further investigation in its role in allergic sensitization.

The commercial IgE test (ImmunoCAP) used in this study was made from cooked edible crabmeat and may therefore not include important allergens such as hemocyanin and enolase. This is also illustrated through comparing the specific IgE to SPT and allergograms. Three king crab workers and six edible crab workers did not have elevated specific IgE to crab, but were SPT positive. Several of the workers who were not SPT positive to one or more of the extracts did show however IgE binding to allergens in the immunoblots. Sensitized crab processing workers may not be diagnosed correctly with the current methods available. A detailed proteomic analysis of all the IgE binding proteins was conducted in this study. IgE binding analysis using a bigger worker cohort to purified novel allergens identified in this study will provide an insight into the presence of novel air-borne food allergens as well as cross reactive allergens responsible for occupational asthma and allergy.

This study is a cross-sectional study design where it is not possible to determine how many workers have left the processing plants due to occupational health problems before the start of the study. The short duration of employment among the crab processing workers and low prevalence of asthma may indicate a healthy worker effect where workers who develop health problems are selected out (59–61). This may result in an underestimation of the true prevalence of occupational health problems among crab processing workers. It causes an underestimation of the health effects of working in the plant. This phenomenon has been discussed in a previous publication on this work population (25). The healthy worker effect may also be an important factor in the lack of a significant association between sensitization to crab and self-reported respiratory symptoms. Our findings may indicate that workers are sensitized to crab, but they have not yet developed asthma or allergy. Sensitization and upper respiratory symptoms often precede the development of asthma and allergy so it may be that when workers develop respiratory symptoms, they leave their job and therefore would not be included in our study.

## Conclusions

Crab processing workers in Norway report respiratory symptoms and are sensitized to specific crab allergens as demonstrated by commercial IgE test, SPT and immunoblotting, and thus are at risk of developing asthma and allergy to crab. Through SPT and immunoblot analysis, we demonstrate that workers in both king crab and edible crab processing are sensitized to at least four allergenic proteins present in both raw and cooked meat, intestines and shell. Two novel occupational crab allergens, enolase and hemocyanin, were identified for the first time. There are several additional unidentified sensitizing allergens to be characterized in the future, enabling better component resolved diagnosis of occupational allergies. Our previous study on air-borne exposure to crab allergens (15) and high prevalence of allergic sensitization to crabs shown in this study, highlight the need to develop improved equipment to protect workers from exposure to bioaerosols. More importantly, improved diagnostic tests, which includes specific seafood species as well as component allergens, are required for better management of occupational asthma and allergy to seafood.

## Data Availability Statement

The datasets presented in this study can be found in online repositories. The names of the repository/repositories and accession number(s) can be found at: https://doi.org/10.25903/qdmh-4r06; https://doi.org/10.25903/78fb-f688.

## Ethics Statement

The studies involving human participants were reviewed and approved by Regional Committee for Medical Research Ethics in Northern Norway. The patients/participants provided their written informed consent to participate in this study.

## Author Contributions

MT helped in conceptualizing the research project, experimental design, data collection in the field, data analysis, manuscript writing, and editing. SK helped in experimental design, data analysis, manuscript writing, and editing. BB was central in conceptualizing the research project, experimental design, data analysis, reviewing, and editing the manuscript. SK and RN helped with the immunoblotting analysis, in gel tryptic digestion, mass spectrometric data analysis, and methods writing. SN and NW performed the proteomic analysis, including experimental work, raw data analysis, writing methods, and provided resources from Bio21 Proteomic Facility. AL helped in conceptualizing the research project, experimental design, reviewing, and editing the manuscript. LA is project manager and was central in conceptualizing the research project, experimental design, reviewing, and editing the manuscript. All authors contributed to the article and approved the submitted version.

## Conflict of Interest

The authors declare that the research was conducted in the absence of any commercial or financial relationships that could be construed as a potential conflict of interest.

## Publisher's Note

All claims expressed in this article are solely those of the authors and do not necessarily represent those of their affiliated organizations, or those of the publisher, the editors and the reviewers. Any product that may be evaluated in this article, or claim that may be made by its manufacturer, is not guaranteed or endorsed by the publisher.
