# Supplementary material for: Occupational Allergic Sensitization Among Workers Processing King Crab (Paralithodes camtschaticus) and Edible Crab (Cancer pagurus) in Norway and Identification of Novel Putative Allergenic Proteins
Source: Front Allergy. 2021 Aug 23;2:718824. doi: 10.3389/falgy.2021.718824 (PMC8974837; doi:10.3389/falgy.2021.718824)
Supplement: Supplementary file 1 [file Table_9.docx]

| **Worker ID** | **Crab** | **Shrimp** | **HDM** | **Atopy status** |
| --- | --- | --- | --- | --- |
|  | ImmunoCAP (kU/L) | ImmunoCAP (‘+’ for >0.35 kU/L, ‘-‘ for <0.35 kU/L) | ImmunoCAP (‘+’ for >0.35 kU/L, ‘-‘ for <0.35 kU/L) |  |
| King crab workers | | | | |
| 1 | 0.55 | - | - | - |
| 2 | 3.31 | - | + | + |
| 3 | 0.59 | - | - | + |
| 4 | 2.62 | - | - | + |
| 5 | 2.39 | + | - | + |
| 6 | 1.76 | + | + | + |
| 7 | 0.89 | - | + | + |
| 8 | 0.67 | - | - | - |
| 9 | 6.61 | - | - | + |
| 10 | 0.38 | - | - | - |
| Edible crab workers | | | | |
| 1 | 1.19 | + | - | - |
| 2 | 1.16 | - | + | + |
| 3 | 0.65 | - | - | + |
| 4 | 14.7 | - | - | + |
| 5 | 5.41 | - | - | + |
| 6 | 0.63 | - | - | - |
| 7 | 4.32 | - | + | + |
| 8 | 3.84 | - | - | + |
| 9 | 11.70 | + | + | + |
| 10 | 105 | - | - | + |

**Supplementary table 9 –** Details on the serum-based allergen-specific IgE quantification for crab processing worker serum samples that were further analysed by IgE immunoblotting against four different crab extracts
