# Supplementary material for: Occupational Allergic Sensitization Among Workers Processing King Crab (Paralithodes camtschaticus) and Edible Crab (Cancer pagurus) in Norway and Identification of Novel Putative Allergenic Proteins
Source: Front Allergy. 2021 Aug 23;2:718824. doi: 10.3389/falgy.2021.718824 (PMC8974837; doi:10.3389/falgy.2021.718824)
Supplement: Supplementary file 2 [file Data_Sheet_1.docx]

**Table S1: King crab raw meat extract**, in-gel tryptic digest mass spectrometric identification of IgE binding proteins isolated from SDS-PAGE bands

| **MW of SDS-PAGE band** | **Identified crab protein** | **Theoretical Molecular weight (Da)** | **Mascot score** | **Coverage** | **Species (closest match in database)** |
| --- | --- | --- | --- | --- | --- |
| **97 kDa** | Calcium-transporting ATPase sarcoplasmic/endoplasmic reticulum | 110272.73 | 71.95 | 0.90% | Artemia franciscana (Brine shrimp) |
|  | Filamin-A | 84.81 | 239031.81 | 1.13% | Drosophila melanogaster (Fruit fly) |
|  | Fructose-bisphosphate aldolase | 72.05 | 39023.31 | 2.22% | Fructose-bisphosphate aldolas |
|  |  |  |  |  |  |
| **75 kDa** | Tropomyosin | 32886.65 | 406.92 | 22.18% | Homarus americanus (American lobster) |
|  | Alpha-actinin | 106459.77 | 119.51 | 1.95 | Anopheles gambiae (African malaria mosquito). |
|  | Hemocyanin C chain | 75826.09 | 54.51 | 1.06 | Panulirus interruptus (California spiny lobster) |
|  | Heat shock 70 kDa protein cognate | 72216.45 | 306.43 | 8.08% | Drosophila melanogaster (Fruit fly) |
|  | 78 kDa glucose-regulated protein | 71876.15 | 176.15 | 6.76% | Echinococcus granulosus (Hydatid tapeworm). |
|  |  |  |  |  |  |
| **65 kDa** | Alpha-actinin | 106459.77 | 82.59 | 1.19% | Anopheles gambiae (African malaria mosquito) |
|  | Tropomyosin | 32817.58 | 61.64 | 4.58% | Blattella germanica (German cockroach) |
|  | Pseudohemocyanin-1 | 79570.32 | 61.46 | 1.61% | Homarus americanus (American lobster |
|  |  |  |  |  |  |
| **48 kDa** | Enolase | 47011.76 | 902.33 | 27.25% | Homarus gammarus (European lobster) |
|  | Actin | 20789.82 | 283.55 | 30.21% | Chionoecetes opilio (Snow Crab) |
|  | Arginine kinase | 40289.38 | 145.45 | 8.96% | Callinectes sapidus (Blue crab) |
|  |  |  |  |  |  |
| **44 kDa** | Actin | 20789.82 | 648.27 | 46.35% | Chionoecetes opilio (Snow Crab) |
|  | Enolase | 47011.76 | 556.53 | 20.09% | Homarus gammarus (European lobster) |
|  | Arginine kinase | 40289.38 | 308.33 | 17.09% | Callinectes sapidus (Blue crab) |
|  |  |  |  |  |  |
| **36 kDa** | Arginine kinase | 40211.34 | 1184.73 | 37.54% | Carcinus maenas (Common shore crab) |
|  | Tropomyosin | 32886.65 | 793.84 | 36.62% | Homarus americanus (American lobster) |
|  | Actin | 20789.82 | 418.51 | 39.06% | Chionoecetes opilio (Snow Crab) |
|  | Glyceraldehyde-3-phosphate dehydrogenase | 35701.2 | 225.43 | 12.01% | Panulirus versicolor (Painted spiny lobster) |
|  |  |  |  |  |  |
| **32 kDa** | Glyceraldehyde-3-phosphate dehydrogenase | 35701.2 | 453.77 | 23.42% | Panulirus versicolor (Painted spiny lobster) |
|  | Arginine kinase | 40211.34 | 423.69 | 19.89% | Carcinus maenas (Common shore crab) |
|  | Tropomyosin | 32817.58 | 208.11 | 10.92% | Blattella germanica (German cockroach) |
|  |  |  |  |  |  |
| **26 kDa** | 14-3-3 protein zeta | 28079.01 | 185.31 | 14.17% | Bombyx mori (Silk moth) |
|  | Troponin I | 23475.67 | 169.35 | 7.96% | Astacus leptodactylus (Turkish narrow-clawed crayfish) |
|  | Actin, acrosomal process isoform | 41747.7 | 108.22 | 4.52% | Limulus polyphemus (Atlantic horseshoe crab) |
|  | Triosephosphate isomerase | 21864.25 | 94.95 | 13.59% | Anopheles merus (Mosquito) |
|  |  |  |  |  |  |
| **15 kDa** | Ubiquitin-conjugating enzyme E2-17 kDa | 16667.44 | 153.2 | 16.33% | Drosophila melanogaster (Fruit fly). |

**Table S2: King crab cooked meat extract**, in-gel tryptic digest mass spectrometric identification of IgE binding proteins isolated from SDS-PAGE bands

| **MW of SDS-PAGE band** | **Identified crab protein** | **Theoretical Molecular weight (Da)** | **Mascot score** | **Coverage** | **Species (closest match in database)** |
| --- | --- | --- | --- | --- | --- |
| **97 kDa** | Myosin heavy chain, muscle | 224327.6 | 522.57 | 4.43% | Drosophila melanogaster (Fruit fly) |
|  | Tropomyosin | 32886.7 | 416.82 | 22.18% | Homarus americanus (American lobster) |
|  | Troponin I | 23475.6 | 61.07 | 3.48% | Astacus leptodactylus (Turkish narrow-clawed crayfish) |
|  |  |  |  |  |  |
| **75 kDa** | Tropomyosin, slow-tonic isoform | 32655.4 | 368.03 | 17.25% | Chionoecetes opilio (Crab |
|  | Myosin heavy chain, muscle | 224327.6 | 208.56 | 1.73% | Drosophila melanogaster (Fruit fly). |
|  |  |  |  |  |  |
| **65 kDA** | Tropomyosin, slow-tonic isoform | 32655.4 | 517.74 | 23.59% | Chionoecetes opilio (Crab |
|  | Myosin heavy chain, muscle | 224327.6 | 203.79 | 1.78% | Drosophila melanogaster (Fruit fly). |
|  | Protein disulfide-isomerase | 32451.3 | 59.6 | 3.23% | Drosophila melanogaster (Fruit fly) |
|  |  |  |  |  |  |
| **48 kDa** | Tropomyosin | 30417.3 | 340.01 | 17.80% | Charybdis feriata (Crucifix crab) |
|  | Rab3 GTPase-activating protein regulatory subunit | 150911.8 | 53.1 | 0.89% | Drosophila melanogaster (Fruit fly) |
|  |  |  |  |  |  |
| **44 kDa** | Tropomyosin | 30417.3 | 333.19 | 17.80% | Charybdis feriata (Crucifix crab) |
|  | Troponin I | 23475.6 | 98.24 | 6.97% | Astacus leptodactylus (Turkish narrow-clawed crayfish) |
|  | Rab3 GTPase-activating protein regulatory subunit | 150911.8 | 53.04 | 0.89% | Drosophila melanogaster (Fruit fly) |
|  |  |  |  |  |  |
| **36 kDa** | Tropomyosin | 31685.9 | 749.9 | 35.40% | Metapenaeus ensis (Greasyback shrimp) |
|  | Fructose-bisphosphate aldolase | 39023.3 | 60.83 | 2.22% | Drosophila melanogaster (Fruit fly) |
|  | Arginine kinase | 40211.3 | 238.58 | 10.36% | Carcinus maenas (Common shore crab) |
|  |  |  |  |  |  |
| **32 kDa** | Tropomyosin | 32886.6 | 824.7 | 37.32% | Homarus americanus (American lobster) |
|  | Glyceraldehyde-3-phosphate dehydrogenase | 35701.2 | 221.07 | 12.01% | Panulirus versicolor (Painted spiny lobster) |
|  | L-lactate dehydrogenase | 35514.5 | 119.59 | 6.93% | Drosophila melanogaster (Fruit fly) |
|  | Arginine kinase | 40211.3 | 100.1 | 4.76% | Carcinus maenas (Common shore crab) |
|  |  |  |  |  |  |
| **26 kDa** | Tropomyosin | 31685.91 | 690.78 | 32.12% | Metapenaeus ensis (Greasyback shrimp) |
|  | Troponin I | 30062.95 | 139.45 | 8.18% | Drosophila melanogaster (Fruit fly) |

**Table S3: King crab intestine extract**, in-gel tryptic digest mass spectrometric identification of IgE binding proteins isolated from SDS-PAGE bands

| **MW of SDS-PAGE band** | **Identified crab protein** | **Theoretical Molecular weight (Da)** | **Mascot score** | **Coverage** | **Species (closest match in database)** |
| --- | --- | --- | --- | --- | --- |
| **97 kDa** | Hemocyanin C chain | 75826.09 | 98.59 | 1.06% | Panulirus interruptus (California spiny lobster) |
|  | D-amino acid oxidase | 36094.58 | 61.72 | 2.17% | Caenorhabditis elegans |
| **75 kDa** | Hemocyanin C chain | 75826.09 | 101.5 | 1.97% | Panulirus interruptus (California spiny lobster) |
|  |  |  |  |  |  |
| **65 kDa** | Myosin heavy chain | 224327.6 | 98.91 | 1.48% | Drosophila melanogaster (Fruit fly) |
|  |  |  |  |  |  |
| **48 kDa** | Enolase | 47011.76 | 248.01 | 9.70% | Homarus gammarus (European lobster) |
|  | Myosin heavy chain | 224327.6 | 204.55 | 1.58% | Drosophila melanogaster (Fruit fly) |
|  |  |  |  |  |  |
| **44 kDa** | Enolase | 47396.14 | 133.31 | 5.53% | Doryteuthis pealeii (Longfin inshore squid) |
|  | Sarcoplasmic calcium-binding protein | 841.5 | 61.49 | 100% | Chionoecetes opilio (Crab-beetle) |
|  |  |  |  |  |  |
| **36 kDa** | Myosin heavy chain | 224327.6 | 217.49 | 1.58% | Drosophila melanogaster (Fruit fly) |
|  | Tropomyosin | 32817.58 | 97.93 | 4.58% | (German cockroach) (Blatta germanica) |
|  | Peptidase 1 | 33199.1 | 38.9 | 3.11% | Psoroptes ovis (Sheep scab mite) |
|  | Vitellogenin | 282850 | 38.42 | 0.35% | Fenneropenaeus merguiensis (Banana prawn) |
|  | Egg-lysin | 15310.7 | 37.03 | 5.13% | Haliotis cracherodii (Black abalone) |
|  |  |  |  |  |  |
| **32 kDa** | Actin | 41814.86 | 158.6 | 7.45% | Lytechinus pictus (Painted sea urchin) |
|  | Tropomyosin | 32817.58 | 143.07 | 10.92% | Blattella germanica (German cockroach) |
|  | Tropomyosin | 32880.64 | 66.98 | 6.34% | Dermatophagoides pteronyssinus (European house dust mite) |
|  | Sarcoplasmic calcium-binding protein | 841.5 | 60.65 | 100% | Chionoecetes opilio (Crab-beetle) |
|  |  |  |  |  |  |
| **26 kDa** | Vitellogenin | 282850 | 49.27 | 0.35% | Fenneropenaeus merguiensis (Banana prawn) |
|  |  |  |  |  |  |
| **15 kDa** | Peptidase 1 | 36227.18 | 39.18 | 3.11% | Psoroptes ovis (Sheep scab mite) |
|  | Phosphoenolpyruvate carboxykinase | 71083.64 | 36.21 | 1.24% | Drosophila melanogaster (Fruit fly) |

**Table S4: King crab shell extract**, in-gel tryptic digest mass spectrometric identification of IgE binding proteins isolated from SDS-PAGE bands

| **MW of SDS-PAGE band** | **Identified crab protein** | **Theoretical Molecular weight (Da)** | **Mascot score** | **Coverage** | **Species (closest match in database)** |
| --- | --- | --- | --- | --- | --- |
|  |  |  |  |  |  |
| **97 kDa** | Gelsolin | 83469.16 | 259.67 | 6.63% | Homarus americanus (American lobster) |
|  | Myosin heavy chain | 224327.62 | 201.27 | 1.48% | Drosophila melanogaster (Fruit fly) |
|  | Paramyosin | 102276.97 | 55.95 | 0.80% | Drosophila melanogaster (Fruit fly) |
|  | Calcium-transporting ATPase sarcoplasmic/endoplasmic reticulum type | 110272.73 | 42.24 | 0.70% | Artemia franciscana (Brine shrimp) |
|  |  |  |  |  |  |
| **75 kDa** | Heat shock 70 kDa protein cognate 3 | 72216.45 | 132.6 | 3.05% | Drosophila melanogaster (Fruit fly) |
|  | Hemocyanin C chain | 75826.09 | 100.84 | 1.97% | Panulirus interruptus (California spiny lobster) |
|  | Hemocyanin A chain | 75647.92 | 88.67 | 1.98% | Panulirus interruptus (California spiny lobster) |
|  | Thioredoxin reductase 1 | 83185.34 | 47.79 | 1.05% | Caenorhabditis elegans |
|  | Phenoloxidase subunit 1 | 78734.69 | 38.15 | 0.88% | Bombyx mori (Silk moth) |
|  | Vitellogenin | 282850.01 | 35.22 | 0.35% | Fenneropenaeus merguiensis (Banana prawn) |
|  |  |  |  |  |  |
| **65 kDa** | Heat shock 70 kDa protein cognate 3 | 72216.45 | 535.33 | 13.26% | Drosophila melanogaster (Fruit fly) |
|  | Hemocyanin B chain | 65247.92 | 186.23 | 5.12% | Astacus leptodactylus (Turkish narrow-clawed crayfish) |
|  | Hemocyanin | 75626.41 | 183.28 | 4.41% | Palinurus vulgaris (European spiny lobster) |
|  | Arginine kinase | 40115.34 | 160.72 | 15.45% | Penaeus monodon (Giant tiger prawn) |
|  | Arginine kinase | 39965.32 | 159.74 | 15.49% | Penaeus japonicus (Kuruma prawn) |
|  | Hemocyanin C chain | 75826.09 | 142.14 | 1.97% | Panulirus interruptus (California spiny lobster) |
|  | Tropomyosin | 32817.58 | 106.21 | 4.58% | Blattella germanica (German cockroach) |
|  |  |  |  |  |  |
| **48 kDa** | No crab protein identified |  |  |  |  |
|  |  |  |  |  |  |
| **44 kDa** | Enolase | 47011.76 | 506.36 | 18.01% | Homarus gammarus (European lobster) |
|  | Actin | 20789.82 | 421.59 | 35.42% | Chionoecetes opilio (Crab-beetle) |
|  | Gelsolin | 83469.16 | 167.12 | 4.24% | Homarus americanus (American lobster) |
|  | Tropomyosin | 32817.58 | 114.38 | 4.58% | Blattella germanica (German cockroach) |
|  |  |  |  |  |  |
| **36 kDa** | Arginine kinase | 40211.34 | 319.8 | 20.73% | Carcinus maenas (Common shore crab) |
|  | Enolase | 47011.76 | 223.39 | 9.70% | Homarus gammarus (European lobster) |
|  | Glutamine synthetase | 40741.96 | 124.28 | 4.43% | Panulirus argus (Caribbean spiny lobster) |
|  | Tropomyosin | 32886.65 | 798.14 | 31.69% | Homarus americanus (American lobster) |
|  | Actin | 20789.82 | 688.57 | 63.02% | Chionoecetes opilio (Crab-beetle) |
|  | Arginine kinase | 40115.34 | 677.35 | 15.17% | Penaeus monodon (Giant tiger prawn) |
|  |  |  |  |  |  |
| **32 kDa** | No crab proteins identified |  |  |  |  |
|  |  |  |  |  |  |
| **26 kDa** | Actin | 20789.82 | 500.77 | 42.19% | Chionoecetes opilio (Crab-beetle) |
|  | Tropomyosin | 32655.48 | 353.11 | 16.90% | Chionoecetes opilio (Crab-beet) |
|  | ADP,ATP carrier protein 1 | 32799.13 | 278.66 | 13.29% | Anopheles gambiae (African malaria mosquito) |
|  | Arginine kinase | 39965.32 | 151.74 | 6.76% | Penaeus japonicus (Kuruma prawn) |
|  | Troponin I | 23475.67 | 101.8 | 6.97% | Astacus leptodactylus (Turkish narrow-clawed crayfish) |
|  | Superoxide dismutase | 15494.77 | 51.74 | 6.94% | Palinurus vulgaris (European spiny lobster) |
|  |  |  |  |  |  |
| **15 kDa** | Actin | 20789.82 | 188.68 | 20.31% | Chionoecetes opilio (Crab-beetle) |
|  | Cofilin/actin-depolymerizing factor homolog | 17142.48 | 66.23 | 7.43% | Drosophila melanogaster (Fruit fly) |
|  | Nucleoside diphosphate kinase B | 6307.59 | 59.43 | 16.67% | Merluccius capensis (Shallow-water Cape hake) |

**Table S5: Edible crab raw meat extract**, in-gel tryptic digest mass spectrometric identification of IgE binding proteins isolated from SDS-PAGE bands

| **MW of SDS-PAGE band** | **Identified crab protein** | **Theoretical Molecular weight (Da)** | **Mascot score** | **Coverage** | **Species (closest match in database)** |
| --- | --- | --- | --- | --- | --- |
|  |  |  |  |  |  |
| **90 kDa** | Hemocyanin | 75626.4 | 186.6 | 4.41% | Palinurus vulgaris (European spiny lobster) |
|  | Alpha-actinin, sarcomeric | 106459.7 | 101.68 | 1.19% | Anopheles gambiae (African malaria mosquito). |
|  | Moesin/ezrin/radixin homolog 1 | 69024.1 | 66.59 | 1.72% | Aedes aegypti (Yellowfever mosquito) (Culex aegypti). |
|  |  |  |  |  |  |
| **74 kDa** | Hemocyanin | 75626.4 | 318.21 | 4.41% | Palinurus vulgaris (European spiny lobster). |
|  |  |  |  |  |  |
| **62 kDa** | Heat shock 70 kDa protein | 71423.5 | 336.25 | 9.17% | Hydra vulgaris (Hydra) |
|  | Hemocyanin | 106951.7 | 325.07 | 5.95% | Palinurus vulgaris (European spiny lobster) |
|  | Alpha-actinin, sarcomeric; | 75826 | 215.41 | 3.33% | Drosophila melanogaster (Fruit fly) |
|  |  |  |  |  |  |
| **50 kDa** | Actin, muscle | 20789.8 | 954.36 | 68.75% | Chionoecetes opilio (Crab-beetle) |
|  | Enolase | 47011.7 | 269.58 | 11.09% | Homarus gammarus (European lobster) |
|  | Arginine kinase | 39965.3 | 245.56 | 10.99% | Penaeus japonicus (Kuruma prawn) |
|  | 26S protease regulatory subunit 8 | 45828.1 | 212.5 | 7.16% | Drosophila melanogaster (Fruit fly) |
|  |  |  |  |  |  |
| **36 kDa** | Arginine kinase | 40211.3 | 1153.7 | 42.86% | Carcinus maenas (Common shore crab) |
|  | Actin, muscle | 20789.8 | 566.62 | 44.79% | Chionoecetes opilio (Crab-beetle) |
|  | Tropomyosin | 32886.6 | 413.57 | 19.01% | Homarus gammarus (European lobster) |
|  |  |  |  |  |  |
| **33 kDa** | Arginine kinase | 40211.3 | 870.15 | 35.01% | Carcinus maenas (Common shore crab) |
|  | Glyceraldehyde-3-phosphate dehydrogenase | 35701.2 | 812.9 | 39.64% | Panulirus versicolor (Painted spiny lobster) |
|  | Fructose-bisphosphate aldolase | 39023.3 | 64.32 | 2.22% | Drosophila melanogaster (Fruit fly) |
|  |  |  |  |  |  |
| **26 kDa** | No protein found related to crab |  |  |  |  |
|  |  |  |  |  |  |
| **19 kDa** | Sarcoplasmic calcium-binding protein, alpha-B and -A chains | 21965.6 | 116.15 | 8.85% | Penaeus sp. (Penoeid shrimp) |
|  |  |  |  |  |  |
| **14 kDa** | No protein found related to crab |  |  |  |  |
|  |  |  |  |  |  |
| **12 kDa** | No protein found related to crab |  |  |  |  |
|  |  |  |  |  |  |

**Table S6: Edible crab cooked meat extract**, in-gel tryptic digest mass spectrometric identification of IgE binding proteins isolated from SDS-PAGE bands

| **MW of SDS-PAGE band** | **Identified crab protein** | **Theoretical Molecular weight (Da)** | **Mascot score** | **Coverage** | **Species (closest match in database)** |
| --- | --- | --- | --- | --- | --- |
|  |  |  |  |  |  |
| **74 kda** | Tropomyosin | 32817.5 | 168.45 | 7.75% | Blattella germanica (German cockroach) |
|  |  |  |  |  |  |
| **62 kDa** | No crab proteins identified |  |  |  |  |
|  |  |  |  |  |  |
| **50 kDa** | Arginine kinase | 40211.3 | 373.09 | 15.41% | Carcinus maenas (Common shore crab) |
|  |  |  |  |  |  |
|  | Tropomyosin | 32655.4 | 1191.91 | 40.85% | Chionoecetes opilio (Snow crab) |
|  |  |  |  |  |  |
| **36 kDa** | Tropomyosin | 32655.4 | 1414.61 | 48.24% | Chionoecetes opilio (Snow crab) |
|  |  |  |  |  |  |
| **33kDa** | Tropomyosin | 32655.4 | 1162.1 | 40.49% | Chionoecetes opilio (Snow crab) |
|  |  |  |  |  |  |
| **26 kDa** | Tropomyosin | 32655.4 | 437.81 | 12.68% | Chionoecetes opilio (Snow crab) |
|  | Tropomyosin | 32886.6 | 708.85 | 28.87% | Homarus americanus (American lobster) |
|  | Troponin I | 23475.6 | 286.91 | 15.92% | Astacus leptodactylus (Turkish narrow-clawed crayfish) |
|  |  |  |  |  |  |
| **19 kDa** | Myosin light chain Flags: Fragments | 1869.8 | 49.35 | 50% | Penaeus monodon (Giant tiger prawn) |
|  |  |  |  |  |  |
| **14 kDa** | Cuticle protein CP1243; | 12419.9 | 424.04 | 79.49% | Cancer pagurus (Rock crab) |
|  | Cuticle protein CP1499 | 14978.7 | 343.67 | 32.43% | Cancer pagurus (Rock crab) |
|  |  |  |  |  |  |
| **12 kDa** | No crab proteins identified |  |  |  |  |
|  |  |  |  |  |  |

**Table S7: Edible crab intestine extract**, in-gel tryptic digest mass spectrometric identification of IgE binding proteins isolated from SDS-PAGE bands

| **MW of SDS-PAGE band** | **Identified crab protein** | **Theoretical Molecular weight (Da)** | **Mascot score** | **Coverage** | **Species (closest match in database)** |
| --- | --- | --- | --- | --- | --- |
| **90 kDa** | No crab proteins identified |  |  |  |  |
|  |  |  |  |  |  |
| **74 kDa** | Hemocyanin subunit B; Flags: Fragment | 4218.1 | 111.18 | 35.14% | Cancer pagurus (Rock crab) |
|  |  |  |  |  |  |
| **62 kDa** | Hemocyanin | 75626.4 | 141.74 | 4.41% | Palinurus vulgaris (European spiny lobster) |
|  |  |  |  |  |  |
| **50 kDa** | Hemocyanin | 75626.4 | 80.65 | 3.50% | Palinurus vulgaris (European spiny lobster) |
|  |  |  |  |  |  |
| **36 kDa** | Actin, cytoskeletal 1 | 41814.8 | 408.71 | 17.55% | Lytechinus pictus (Painted sea urchin) |
|  |  |  |  |  |  |
| **33 kDa** | Tropomyosin | 32817.5 | 176.89 | 7.39% | Blattella germanica (German cockroach) |
|  |  |  |  |  |  |
| **26 kDa** | No crab proteins identified |  |  |  |  |
|  |  |  |  |  |  |
| **19 kDa** | Not tested |  |  |  |  |
|  |  |  |  |  |  |
| **14 kDa** | Not tested |  |  |  |  |
|  |  |  |  |  |  |
| **12 kDa** | Not tested |  |  |  |  |

**Table S8: Edible crab shell extract**, in-gel tryptic digest mass spectrometric identification of IgE binding proteins isolated from SDS-PAGE bands

| **MW of SDS-PAGE band** | **Identified crab protein** | **Theoretical Molecular weight (Da)** | **Mascot score** | **Coverage** | **Species (closest match in database)** |
| --- | --- | --- | --- | --- | --- |
|  |  |  |  |  |  |
| **90 kDa** | Arginine kinase | 40211.34 | 177.44 | 13.73% | Carcinus maenas (Common shore crab) |
|  | Hemocyanin subunit B | 4218.1 | 126.54 | 35.14% | Cancer pagurus (Rock crab) |
|  | Fructose-bisphosphate aldolase | 39023.31 | 53.59 | 2.22% | Drosophila melanogaster (Fruit fly) |
|  | Cuticle protein CP1243 | 12419.94 | 48.06 | 5.13% | Cancer pagurus (Rock crab) |
|  |  |  |  |  |  |
| **74 kDa** | Hemocyanin | 75626.41 | 209.48 | 4.41% | Palinurus vulgaris (European spiny lobster) |
|  | Hemocyanin C chain | 75826.09 | 137.62 | 1.97% | Panulirus interruptus (California spiny lobster) |
|  | Arginine kinase | 40211.34 | 115.55 | 10.92% | Carcinus maenas (Common shore crab) |
|  |  |  |  |  |  |
| **62 kDa** | Hemocyanin | 75626.41 | 221.92 | 4.41% | Palinurus vulgaris (European spiny lobster) |
|  | Hemocyanin C chain | 75826.09 | 164 | 5.60% | Panulirus interruptus (California spiny lobster) |
|  | Heat shock 70 kDa protein A | 69679.64 | 109.78 | 5.78% | Caenorhabditis elegans |
|  | Arginine kinase | 39951.18 | 75.32 | 2.81% | Artemia franciscana (Brine shrimp) |
|  |  |  |  |  |  |
| **50 kDa** | Arginine kinase | 40211.34 | 265.5 | 10.08% | Carcinus maenas (Common shore crab) |
|  | Fructose-bisphosphate aldolase B | 39620.14 | 104.5 | 3.85% | Sparus aurata (Gilthead sea bream) |
|  |  |  |  |  |  |
| **36 kDa** | Tropomyosin | 32655.48 | 1224.66 | 40.85% | Chionoecetes opilio (Crab-beet |
|  | Tropomyosin | 32886.65 | 1174.33 | 37.32% | Homarus americanus (American lobster) |
|  | Actin | 41758.85 | 440.39 | 16.76% | Artemia sp. (Brine shrimp) |
|  | Fructose-bisphosphate aldolase | 35379.39 | 76.03 | 3.86% | Danio rerio (Zebrafish) |
|  |  |  |  |  |  |
| **33 kDa** | Tropomyosin | 30417.38 | 714.13 | 35.61% | Charybdis feriata (Crucifix crab) |
|  | Glyceraldehyde-3-phosphate dehydrogenase | 35693.18 | 237.89 | 14.11% | Homarus americanus (American lobster) |
|  | Cuticle protein CP1243 | 12419.94 | 44.37 | 5.13% | Cancer pagurus (Rock crab) |
|  | Fructose-bisphosphate aldolase | 39023.31 | 53.77 | 2.22% | Drosophila melanogaster (Fruit fly) |
|  |  |  |  |  |  |
| **26 kDa** | Not tested |  |  |  |  |
|  |  |  |  |  |  |
| **19 kDa** | Cuticle protein AM1159 | 11578.57 | 91.99 | 11.43% | Cancer pagurus (Rock crab) |
|  | Cuticle protein AM1199 | 12003.89 | 91.99 | 11.11% | Cancer pagurus (Rock crab) |
|  | Cuticle protein CP1243 | 12419.94 | 48.94 | 5.13% | Cancer pagurus (Rock crab) |
|  | Peptidase 1 | 33199.63 | 38.9 | 3.11% | Psoroptes ovis (Sheep scab mite) |
|  |  |  |  |  |  |
| **14 kDa** | Cuticle protein CP1243 | 12419.94 | 225.92 | 46.15% | Cancer pagurus (Rock crab) |
|  | Cuticle protein CP1499 | 14978.76 | 73.05 | 8.11% | Cancer pagurus (Rock crab) |
|  | Titin | 2064498.85 | 68.11 | 68.11 | Drosophila melanogaster (Fruit fly) |
|  |  |  |  |  |  |
| **12 kDa** | Not tested |  |  |  |  |
|  |  |  |  |  |  |
