# Supplementary material for: Occupational Allergic Sensitization Among Workers Processing King Crab (Paralithodes camtschaticus) and Edible Crab (Cancer pagurus) in Norway and Identification of Novel Putative Allergenic Proteins
Source: Front Allergy. 2021 Aug 23;2:718824. doi: 10.3389/falgy.2021.718824 (PMC8974837; doi:10.3389/falgy.2021.718824)

Supplementary Figure 1A

King Crab raw extract (KCR)

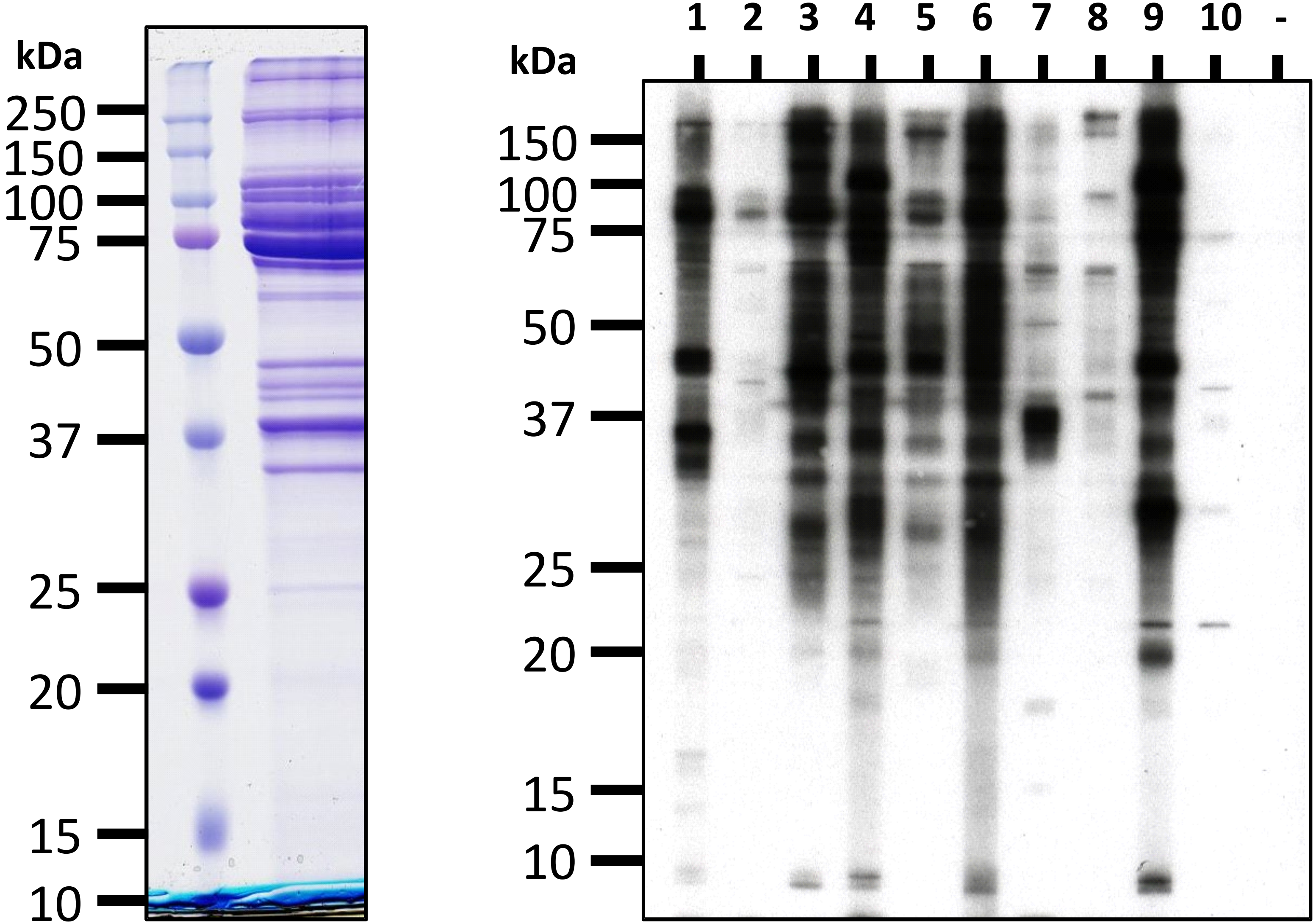

Supplementary Figure 1B

King Crab cooked extract (KCC)

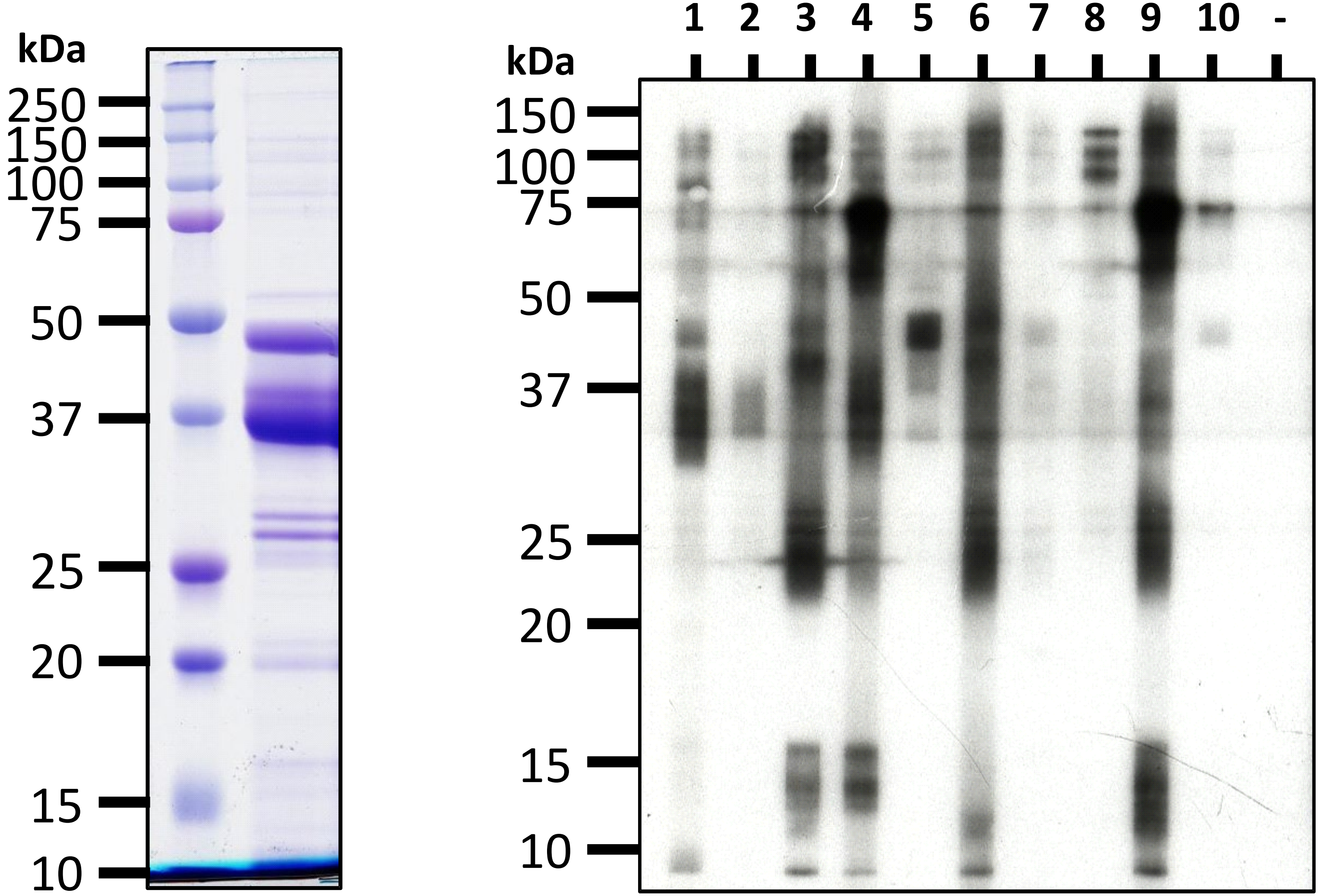

Supplementary Figure 1C

King Crab Intestine extract (KCl)

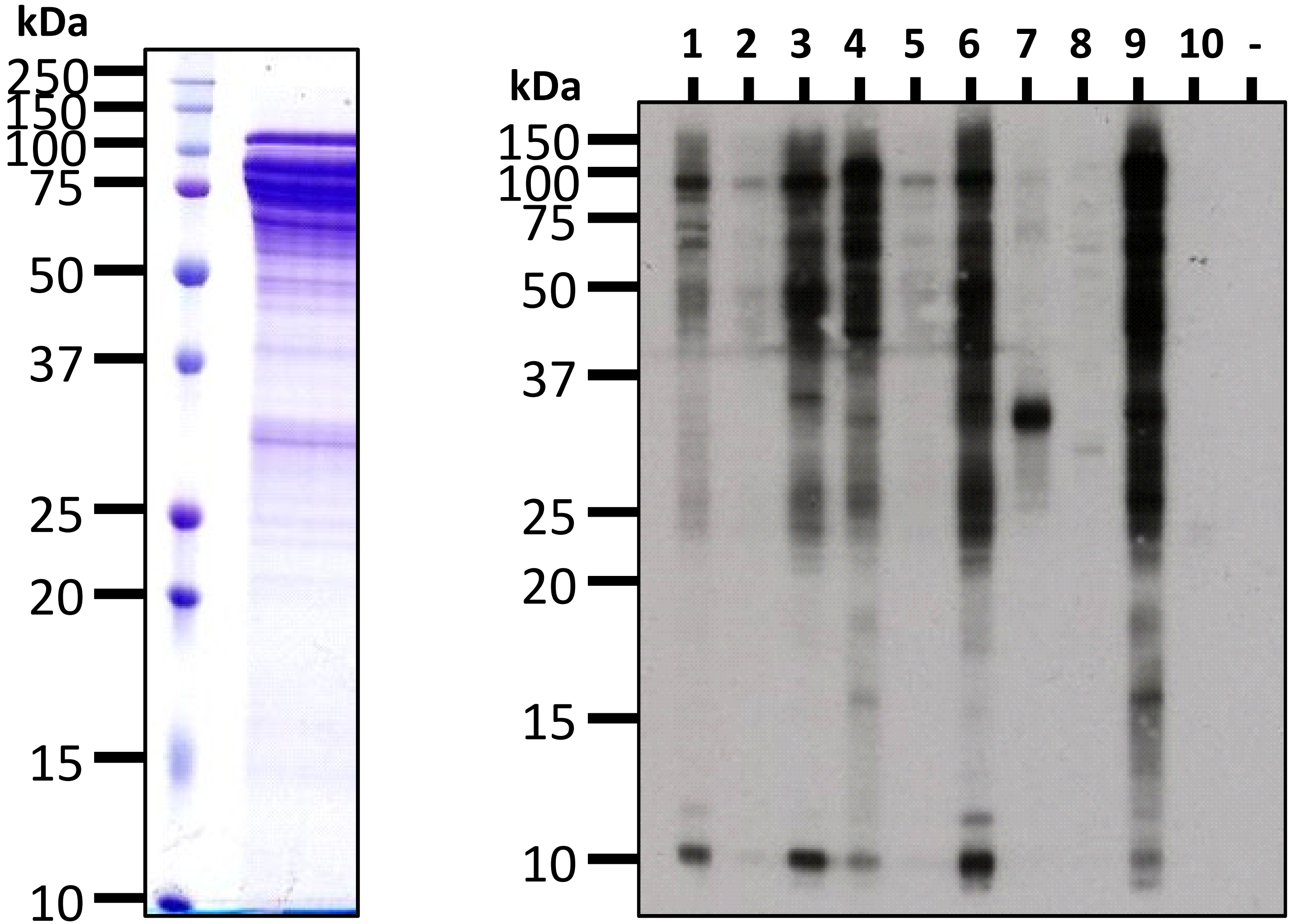

Supplementary Figure 1D

King Crab Shell extract (KCS)

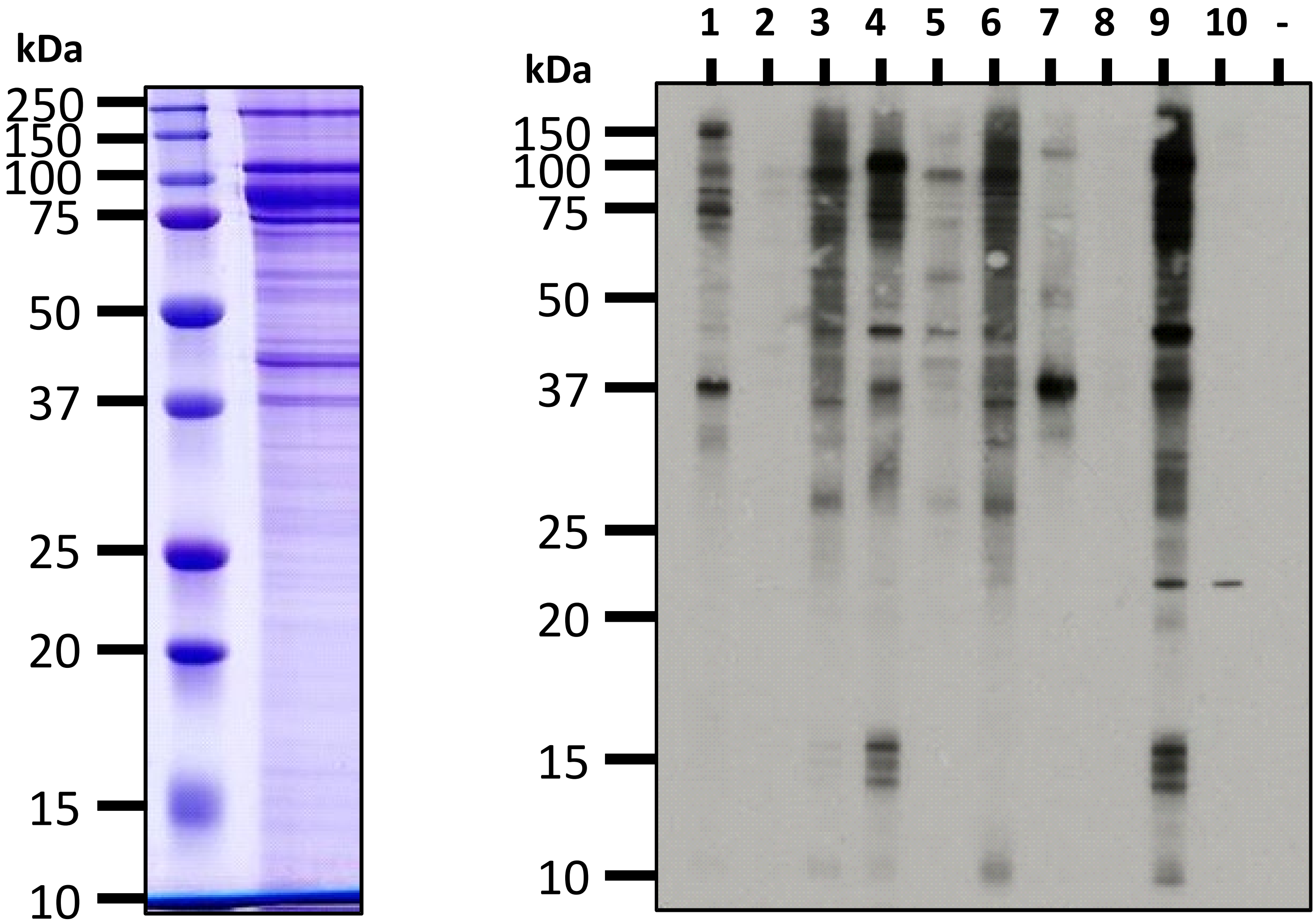

Supplementary Figure 1E

Edible Crab Raw extract (ECR)

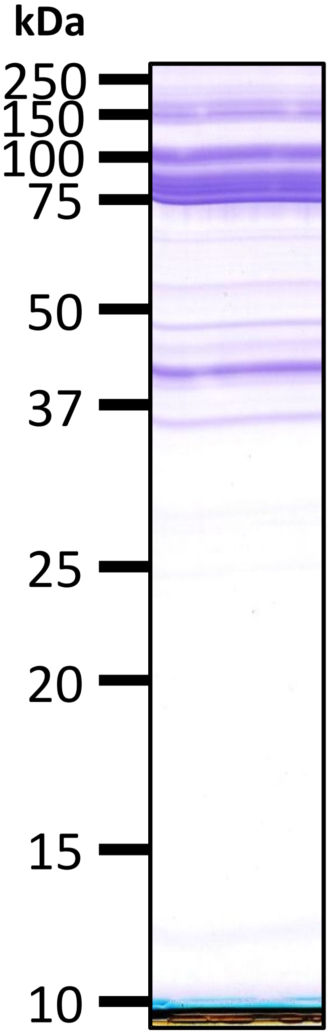

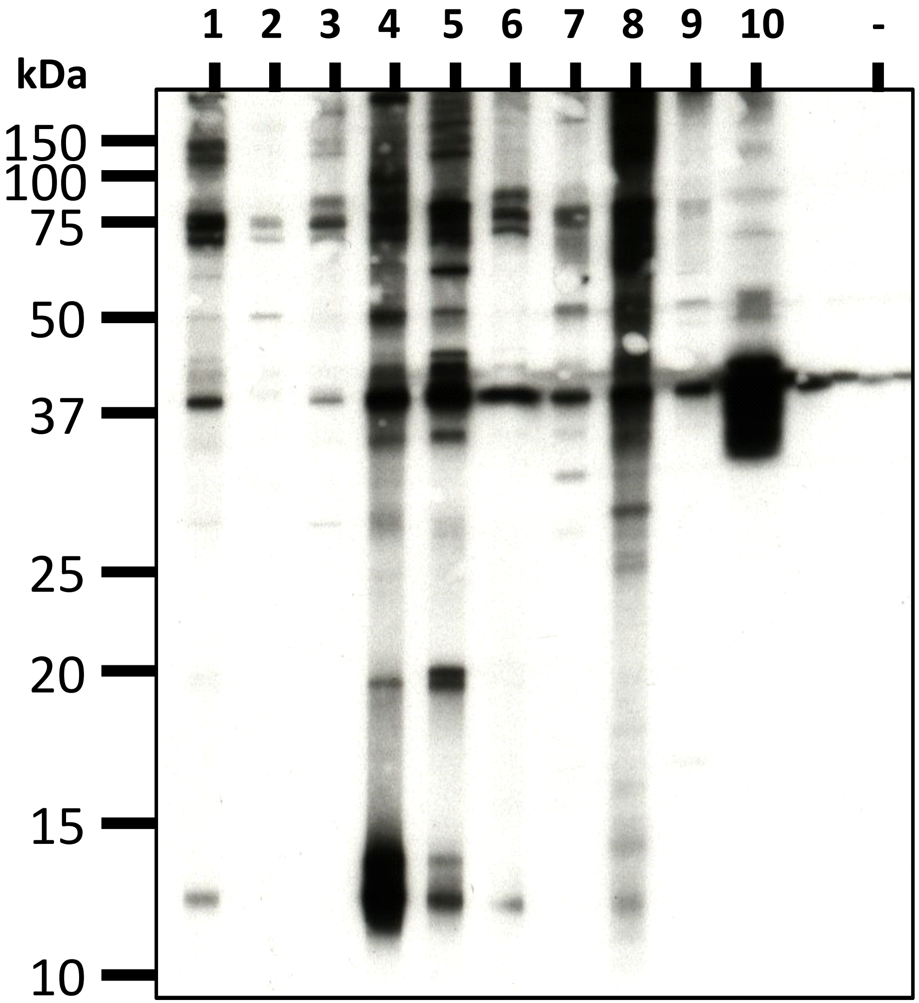

Supplementary Figure 1F

Edible Crab Cooked extract (ECC)

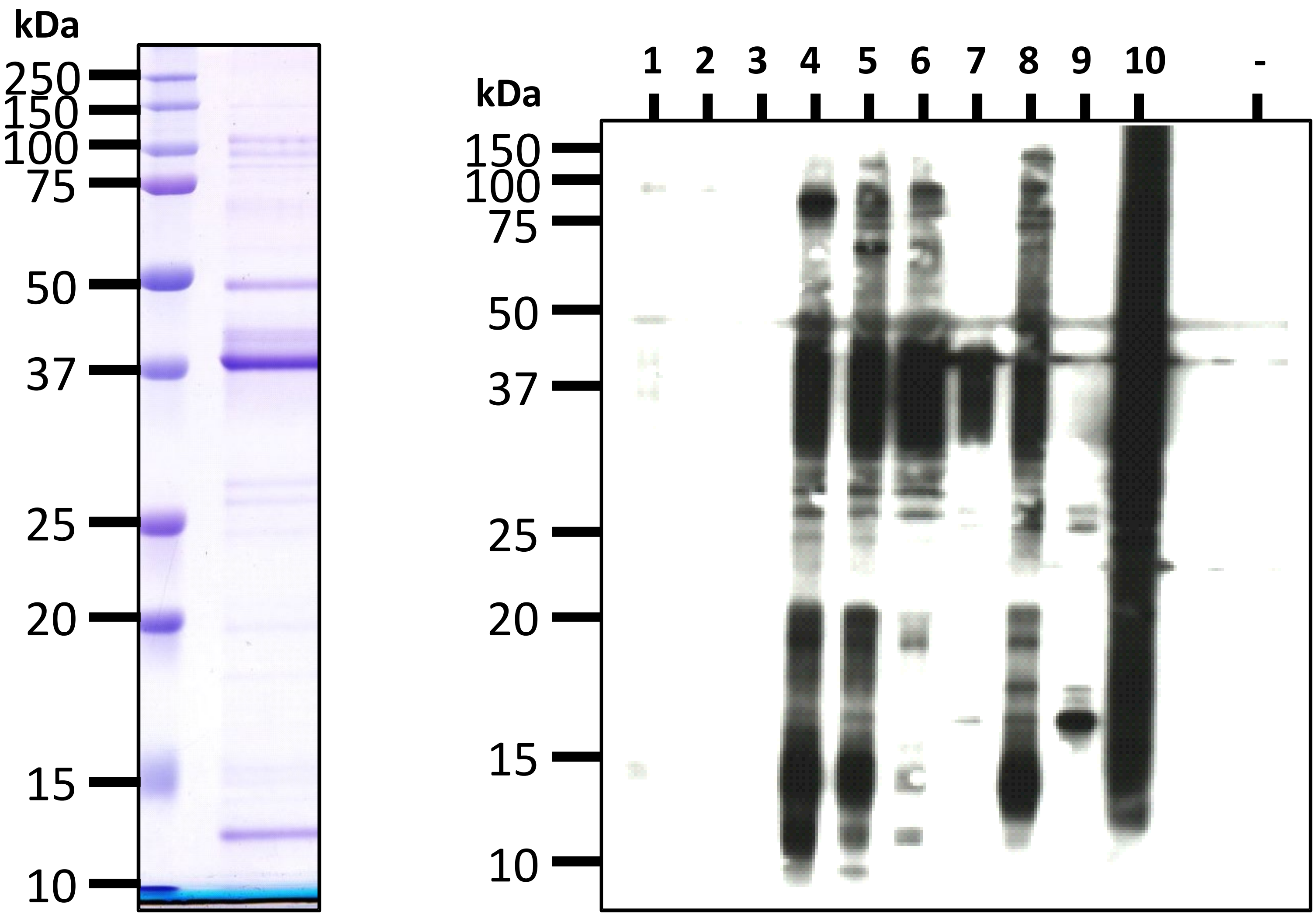

Supplementary Figure 1G

Edible Crab Intestine extract (ECI)

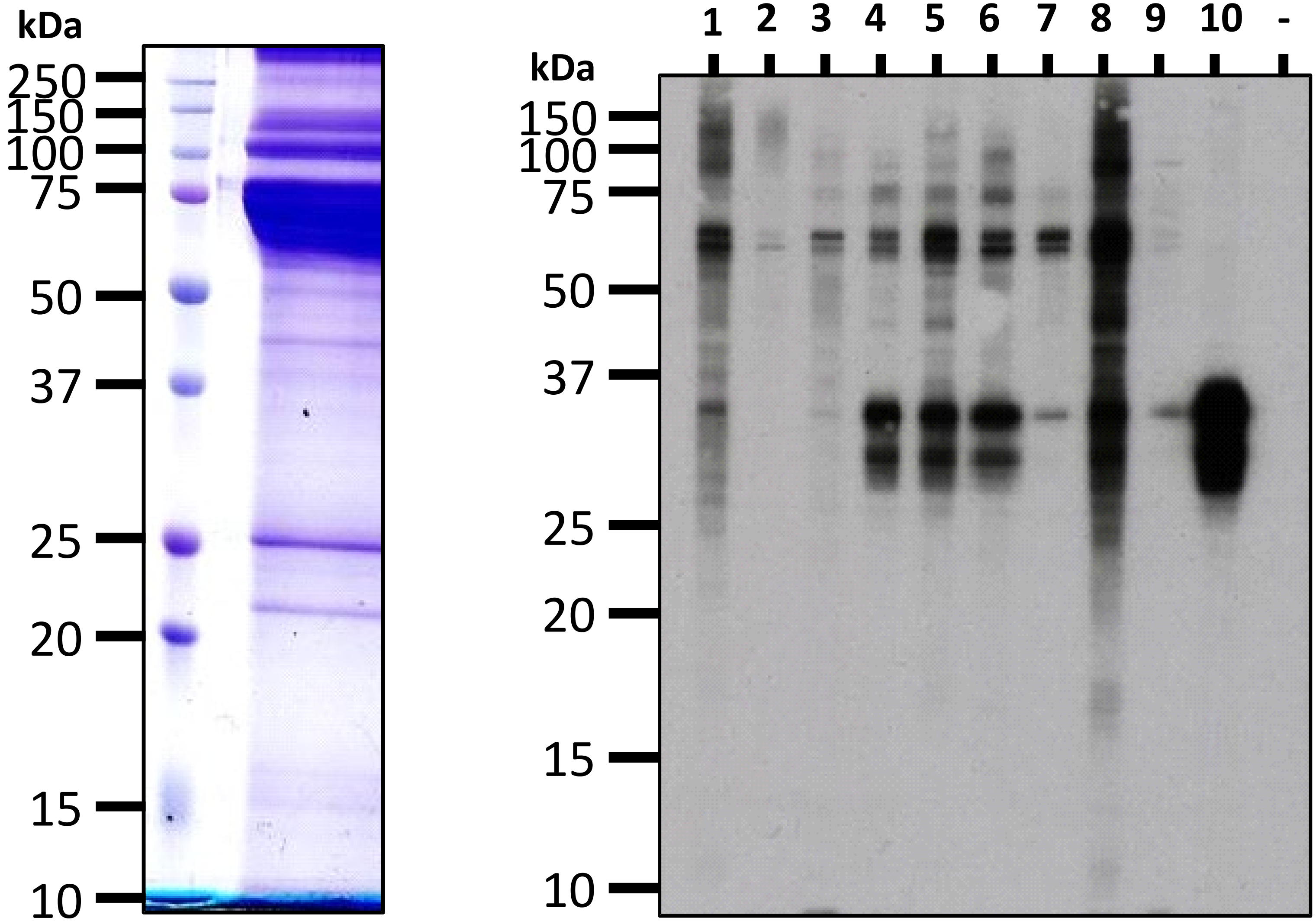

Supplementary Figure 1H

Edible Crab Shell extract (ECS)

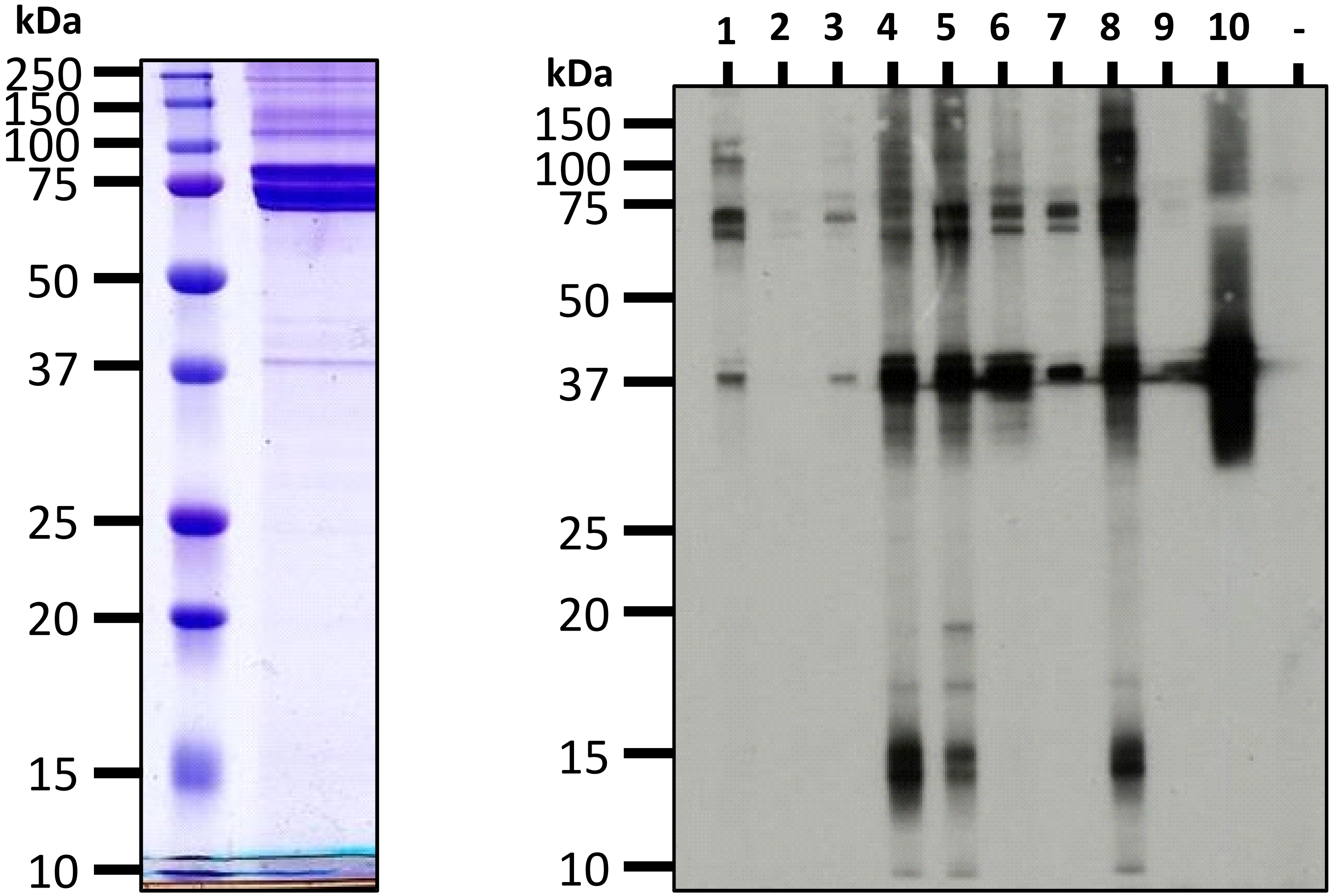

Supplement: Supplementary file 3 [file Data_Sheet_2.pdf]
